# Risk of malignancy and surveillance of premalignant gastrointestinal lesions in adult celiac disease

**DOI:** 10.3389/fonc.2026.1876345

**Published:** 2026-09-03

**Authors:** Jan Bures, Katerina Kamaradova, Vladimira Rezacova, Michaela Zapletalova, Michaela Cvanova, Stepan Suchanek, Pavel Drastich, Nadija Brodyuk, Jiri Dolezal, Tomas Tuma, Jozef Malik, Tomas Grega, Doris Vokurkova, Radek Pohnan, Petr Urbanek, Miroslav Zavoral, Darina Kohoutova

**Affiliations:** 1 Institute of Gastrointestinal Oncology, Military University Hospital, Prague, Czechia; 2 Department of Medicine, Charles University, First Faculty of Medicine, Prague and Military University Hospital, Prague, Czechia; 3 Biomedical Research Centre, University Hospital, Hradec Kralove, Czechia; 4 The Fingerland Department of Pathology, Charles University, Faculty of Medicine in Hradec Kralove; University Hospital, Hradec Kralove, Czechia; 5 Institute of Clinical Immunology and Allergology, University Hospital, Hradec Kralove, Czechia; 6 National Screening Centre, Institute of Health Information and Statistics of the Czech Republic, Prague, Czechia; 7 Institute of Biostatistics and Analyses, Masaryk University, Faculty of Medicine, Brno, Czechia; 8 Department of Hepatogastroenterology, Institute for Clinical and Experimental Medicine, Prague, Czechia; 9 Department of Nuclear Medicine, University Hospital, Hradec Kralove, Czechia; 10 Department of Radiodiagnostics, Military University Hospital, Prague, Czechia; 11 Department of Surgery, Charles University, Second Faculty of Medicine, Prague and Military University Hospital, Prague, Czechia; 12 The Royal Marsden NHS Foundation Trust, London, United Kingdom

**Keywords:** cancer, celiac disease, functional hyposplenism, lymphoma, refractory sprue

## Abstract

Celiac disease is a systemic autoimmune affliction triggered by dietary gluten in genetically predisposed subjects. Current estimation of the prevalence of celiac disease is approximately 1% for the global population. These data are consistent with current figures from the Czech Republic (prevalence of 1.06% in 2023). Celiac disease can be associated with several complications including malignancy. Exact epidemiology data on T-cell lymphoma and small intestinal adenocarcinoma in the Czech Republic over the period 2010–2023 are also provided. Unlike the no-biopsy approach in children, despite current non-conclusive discussion and besides serology, upper gastrointestinal endoscopy, biopsies, and histology are mandatory for proper diagnosis and initial assessment of the severity of celiac disease in adults. However, there are so far no unified and integrated recommendations for the follow-up of adult celiac disease. Although the risk of malignancy in celiac disease is relatively low, especially in a strict gluten-free diet, it might nevertheless be further decreased by an individualized surveillance. Endoscopy controls with biopsies for histology, immunohistochemistry, and flow cytometry are essential. The purpose of this article was to review the risk and diagnostics of malignancy in celiac disease. A proposal for the individualized surveillance of premalignant gastrointestinal lesions in adult celiac disease was drafted.

## 1 Introduction

Celiac disease is a systemic autoimmune affliction triggered by dietary gluten in genetically predisposed subjects (1–4). It consists of at least three related disturbances: “classical” illness (>95%), “ultra-short” celiac disease (∼1%), and seronegative celiac disease (∼3%) (1, 5–9). Several guidelines and reviews on diagnostics and therapy have been published recently (2, 4, 10–20). In contrast to the no-biopsy approach in children (21, 22), despite current non-conclusive discussion (23, 24), notwithstanding serology, upper gastrointestinal endoscopy, biopsies, and histology are mandatory for the proper diagnosis and initial assessment of the severity of celiac disease in adults (25–35) (**Supplementary Figures 1**-**5**). However, there are so far no unified and integrated recommendations for the follow-up of adult celiac disease (36). The purpose of our current article was to review the risk and diagnostics of malignancy in celiac disease. A proposal for the individualized surveillance of premalignant gastrointestinal lesions in adult celiac disease was drafted as the second aim of this paper.

## 2 Monitoring of established celiac disease

Surveillance of established celiac disease is based on the assessment of gluten-free diet adherence, nutritional status, and monitoring for complications. After the first control within 3–4 months, a 2-year follow-up interval in stable patients is recommended (4, 13, 16, 17, 37, 38).

Assessment of adult celiac disease activity has currently not yet been fully solved. Hindryckx et al. (39) published a systematic review of 286 studies. Various indices include symptoms, serology, endoscopy, histology, calprotectin, and several other laboratory markers (e.g., gluten-reactive T-lymphocytes and intestinal fatty acid-binding protein). Celiac Disease Patient-Reported Outcome (CeD PRO) and Celiac Disease Symptom Diary (CDSD) were approved by the Food and Drug administration (FDA) (39, 40).

## 3 Epidemiology of celiac disease and associated gastrointestinal malignancies in the Czech Republic

In the past, prevalence of celiac disease in the Czech Republic was estimated to be 1:250–1:200, including anticipated asymptomatic subjects who had not so far been diagnosed with celiac disease. We now have exact epidemiology data over the period 2010–2023, sourced from the two Czech national registries, namely, the Institute of Health Information and Statistics of the Czech Republic (National Registry of Reimbursed Health Services and National Oncology Registry) and the Czech Statistical Office (population demographics) (see **Tables 1**–**3**). The number of subjects diagnosed with celiac disease has increased substantially over the period 2010–2023. Obligatory national guidelines on celiac disease were introduced into clinical practice of the Czech Republic in 2011 (10). The available data do not allow to state with certainty whether the increase in recognized cases of celiac disease is due to improved diagnostics (likely in the first half of this time interval) or reflects a true rise in prevalence (probably in the second part of the monitoring period). Despite a risk of small-number error, the incidence of both T-cell lymphoma and adenocarcinoma of the small intestine in subjects diagnosed with celiac disease is clearly higher compared to that of the general population. Because of significant fluctuations in the incidence data over several years, the figures must be evaluated with caution to avoid over-interpreting year-to-year variations of these two malignancies.

**Table 1:** Prevalence of celiac disease in the Czech Republic – number of living subjects diagnosed with this disease or its history till 31st December in a given year.

Year	2010	2011	2012	2013	2014	2015	2016	2017	2018	2019	2020	2021	2022	2023
Number	13,310	21,409	29,069	37,066	45,351	53,320	60,580	67,597	74,983	83,765	91,185	98,495	106,849	115,265
Calculation	126.55	203.96	276.60	352.65	430.90	505.74	573.39	638.34	705.63	785.10	852.18	937.97	993.06	1059.61

Number: total number of patients in a given year in the Czech Republic.

Calculation: prevalence per 100,000 general population.

Celiac disease was identified as diagnosis K90.0 according to the WHO International Classification of Diseases (version ICD-10).

**Table 2:** Incidence of T cell lymphoma – number of newly diagnosed subjects in a given year.

Year	2010	2011	2012	2013	2014	2015	2016	2017	2018	2019	2020	2021	2022	2023
No. A	37	59	35	58	52	47	43	50	52	46	37	48	36	50
Count 1	0.35	0.56	0.33	0.55	0.49	0.45	0.41	0.47	0.49	0.43	0.35	0.46	0.33	0.46
No. B	2	0	0	1	1	0	0	2	2	1	0	0	0	0
Count 2	15.03	0	0	2.70	2.21	0	0	2.96	2.67	1.19	0	0	0	0

No. A: total number of subjects newly diagnosed with T cell lymphoma in a given year.

Count 1: incidence of T cell lymphoma per 100,000 general population.

No. B: total number of patients newly diagnosed with T cell lymphoma in subjects with established celiac disease in a given year.

Count 2: incidence of T cell lymphoma per 100,000 patients diagnosed with established celiac disease.

T-cell lymphoma was identified as diagnosis C84.4 according to the WHO International Classification of Diseases (version ICD-10).

**Table 3:** Incidence of adenocarcinoma of the small intestine – number of newly diagnosed subjects in a given year.

Year	2010	2011	2012	2013	2014	2015	2016	2017	2018	2019	2020	2021	2022	2023
No. A	58	52	60	43	63	55	50	61	81	90	84	70	82	95
Count 1	0.55	0.50	0.57	0.41	0.60	0.52	0.47	0.58	0.76	0.84	0.79	0.67	0.76	0.87
No. B	2	1	2	2	1	2	0	2	5	5	3	1	1	9
Count 2	15.03	4.67	6.88	5.40	2.21	3.75	0	2.96	6.67	5.97	3.29	1.02	0.94	7.81

No. A: total number of subjects newly diagnosed with adenocarcinoma of the small intestine in a given year.

Count 1: incidence of adenocarcinoma of the small intestine per 100,000 general population.

No. B: total number of patients newly diagnosed with adenocarcinoma of the small intestine in subjects with celiac disease in a given year.

Count 2: incidence of adenocarcinoma of the small intestine per 100,000 patients diagnosed with celiac disease.

Adenocarcinoma of the small intestine was identified as diagnosis C17.X according to the WHO International Classification of Diseases (version ICD-10) in combination with adenocarcinoma morphology according to ICD-O-3.

## 4 Slow responders to gluten-free diet

Despite 6–12 months of gluten-free diet, some patients with established adult celiac disease (up to one-third of subjects) still have continuing symptoms and/or laboratory abnormalities (13, 41). A slow response to a gluten-free diet is not necessarily considered a persisting pathological process, but rather a delayed recovery of the organism. Adherence to a gluten-free diet should be evaluated first (stool or urine 33-mer gliadin, stool gluten immunogenic peptides) and possible coincident disorders should be considered (e.g., food intolerance or small intestinal bacterial overgrowth) (13, 42). However, normal serology does not fully exclude unintended low-level gluten exposure that can be responsible for persistent disease activity (13). Once dietary and other causes are excluded, duodenal biopsies should be repeated to exclude refractory sprue (13, 24).

## 5 Type I refractory sprue

Refractory celiac disease (refractory sprue) is defined as a persistence or recurrence of symptoms and small intestinal villous atrophy after 12–24 months of a strict gluten-free diet, together with negative (i.e., normalized) serology (16, 24, 43–50). Differentiation between type I and type II refractory sprue is crucial, based on flow cytometry (considered as gold standard) and immunophenotyping (16, 24, 26, 43–50) (see **Supplementary Figures 6**-**9**). Type I has negative or low-rate aberrant intra-epithelial lymphocytes (<20%), showing a fully polyclonal T-cell population. Surface CD3 and CD8 expression on intra-epithelial T lymphocytes is preserved (24, 51). Oral medical therapy for type I refractory sprue includes budesonide, followed optionally by systemic glucocorticoids and azathioprine (first-line treatment); 6-thioguanine is a safe alternative as the second-line treatment (24, 52).

## 6 Type II refractory sprue

Type II refractory celiac disease (refractory sprue) is a serious pre-lymphomatous condition, a true “window to lymphoma” (24, 53). It is characterized by a clonal expansion of aberrant intra-epithelial T lymphocytes (>20%). Expression of CD3 is cytoplasmic only, and expression of CD8 is absent (16, 24, 43, 44, 50, 54–56) (see **Supplementary Figures 10**-**15**). Type II refractory sprue is associated with HLA-DQ2 homozygosity (∼60%) (24). Endoscopic findings of ulcers and/or stenoses are commonly seen (**Supplementary Figure 10**). Diarrhea, severe protein-energy malnutrition, and anemia are typical signs of type II refractory sprue (32). Regular wireless small bowel capsule endoscopy should be considered for these patients (57, 58). Effective medical therapy of type II refractory sprue has not yet been fully set (24). However, tofacitinib (a JAK1/JAK3 inhibitor) and tocilizumab (an interleukin-6 blocker) seem to be a promising option in the future (59–61).

## 7 Enteropathy-associated T-cell lymphoma

A possible connection of celiac disease with lymphoma was first noted by Fairley and Mackie in 1937 (62), who linked steatorrhea to small intestinal lymphoma. Later, in 1962, Gough et al. suggested that celiac disease might be a premalignant condition after describing lymphoma in three patients with long-standing celiac disease (63).

Enteropathy-associated T-cell lymphoma arises from intestinal intraepithelial cytotoxic T cells (64). Risk factors include poor adherence to a gluten-free diet, advanced age, male gender, and HLA-DQ2 homozygosity. The small bowel is involved in more than 90%. Patients typically present with abdominal pain and systemic lymphoma “B symptoms” (low-grade fever, weight loss, and night sweats) (64). Emergency is caused by intestinal perforation and/or life-threatening bleeding untreatable by endoscopy or therapeutic radiology. Diagnostics are based on imaging, either non-invasive (wireless small bowel capsule endoscopy or MR enterography) or invasive (deep enteroscopy and/or arteriography) (57, 65–67) (see **Supplementary Figures 16**-**19**). Patients should not be referred for surgery (apart from in cases of bowel perforation), as the small intestinal involvement is typically multifocal (64). Patients with diagnosed enteropathy-associated T-cell lymphoma are referred to a multidisciplinary team to establish an optimal subsequent therapy.

Enteropathy-associated T-cell lymphoma accounts for about one-third of all lymphomas complicating celiac disease. The remaining subtypes comprise diffuse large B-cell lymphoma (**Supplementary Figure 20**), follicular lymphoma (**Supplementary Figure 21**), other intestinal and non-intestinal mature B-cell lymphomas, and T-cell and NK cell lymphomas (68–70).

## 8 Adenocarcinoma of the small intestine

Small intestinal adenocarcinoma is a relatively rare complication of adult celiac disease (71) (see **Table 3**, **Supplementary Figures 22**, **23**). Its incidence has been stable in the Czech Republic during the past 15 years. It usually involves the proximal jejunum. Major presenting symptoms include abdominal pain, nausea and vomiting, anemia, and/or overt bleeding (71, 72). Imaging workup usually starts with abdominal CT scan, followed by wireless small bowel capsule endoscopy, and/or deep enteroscopy.

Prognosis of enteropathy-associated T-cell lymphoma is much worse compared to adenocarcinoma of the small intestine in subjects previously diagnosed with celiac disease (73, 74). Santacroce et al. (73) evaluated 86 patients over the period 2000–2024. Median survival rate was 14 months for T-cell lymphoma while the median was not reached for small intestinal adenocarcinoma. During a 24-month follow-up, there was a significant difference in lethality rate (76% for enteropathy-associated T-cell lymphoma vs. 12% in adenocarcinoma of the small intestine) (73). Preceding refractory sprue was identified in 30 out of 43 cases (70%) of T-cell lymphoma while only in 1 in 43 cases (4%) with small intestinal adenocarcinoma (73).

## 9 Extraintestinal malignancies in celiac disease

Apart from lymphomas and small intestinal cancer, celiac disease can also be complicated by other malignancies (75–77). They include leukemias, melanoma, and cancers of the esophagus, stomach, liver, thyroid, uterus, prostate, and testis (see Refs (36, 75, 77). for review and details).

Celiac disease is also associated with an increased risk of ductal adenocarcinoma of the pancreas (OR up to 1.8) (36, 76, 78). Pancreatic cancer has a very poor prognosis in general. Surgery is not necessarily curative even in the case of tumor R0 resection of earlier stages (T1 or T2), as micrometastases may already be established. Postoperative bleeding is uncommon but may be a serious complication (**Supplementary Figure 24**).

Gastro-entero-pancreatic neuroendocrine neoplasms (NENs) are rare tumors. Some authors hypothesize that chronic inflammation in celiac disease may play a role in NEN pathogenesis and thus consider reasonable testing for preceding/coexisting celiac disease in NENs (76, 79, 80). Testing for celiac disease could determine if this association is causal or just coincidental. Furthermore, it may clarify how chronic inflammation drives NEN tumorigenesis (80).

## 10 Functional hyposplenism

The prevalence of functional hyposplenism is high in celiac disease, affecting up to one-third of patients (81–84). Splenic function should be investigated in cases of refractory sprue, other concomitant autoimmune disorders, older age at diagnosis of celiac disease, previous history of severe infections or thromboembolism, mesenteric lymph node cavitation, and/or spleen atrophy (85). Appropriate diagnosis is possible thanks to flow cytometry (85, 86) (see **Supplementary Figure 25**). Subjects with severe hyposplenism should be vaccinated against *Streptococcus pneumoniae*, *Neisseria meningitidis*, and *Haemophilus influenzae* type B (81–84). HLA DQ2-positive patients may have an impaired response to vaccines against *Hepatitis B Virus*, and hence, vaccination during clinical remission of celiac disease should be preferred (83). Other vaccines should be considered and recommended on a personalized basis (83).

Although initial studies on hyposplenism did not find an increased risk of malignancy (87), hyposplenism in celiac disease was subsequently shown to be associated with refractory sprue, increased risk of T-cell lymphoma, and other serious complications (e.g., ulcerative jejunoileitis) (88).

## 11 Proposal of personalized surveillance of celiac disease and associated gastrointestinal premalignant conditions in adults

Current guidelines do not provide standardized recommendations for surveillance of celiac disease and diagnostics of premalignant lesions, particularly regarding follow-up intervals, risk stratification, and monitoring strategies for high-risk patients, and especially in cases of refractory sprue of both types (36).

We have proposed an individualized surveillance of celiac disease and refractory sprue in adults(see **Algorithm 1**). This algorithm was decided as a simplified form to keep its comprehensibility. European guidelines (13) provide detailed information on other diagnostic markers (including strength of recommendation and level of evidence), non-consistent data on follow-up, different approach for slow responders to a gluten-free diet, and unequal treatment options of refractory celiac disease (13). From our point of view, essential precondition is an initial endoscopy with biopsies at diagnosis. It enables future assessment of treatment efficacy and/or management of complications. According to our vast experience, even severe small intestinal involvement (Marsh 3c) could be fully normalized within 2 years of strict gluten-free diet (to Marsh 0 or 1). Significant improvement can already be seen after the first 6 months. If not, refractory celiac disease should already be considered after the first control endoscopy and histology. Another important point is to take advantage of immunohistochemistry and flow cytometry to distinguish type I and type II refractory sprue. The main reason is to establish an individualized approach because of different risk of malignancy. However, other factors should be taken into consideration as well, such as anemia, malnutrition, metabolic bone disease, deficiency in other vitamins (outside of vitamin D), functional hyposplenism, and lack of trace elements (3, 4, 13, 17, 24).

**Algorithm 1:**
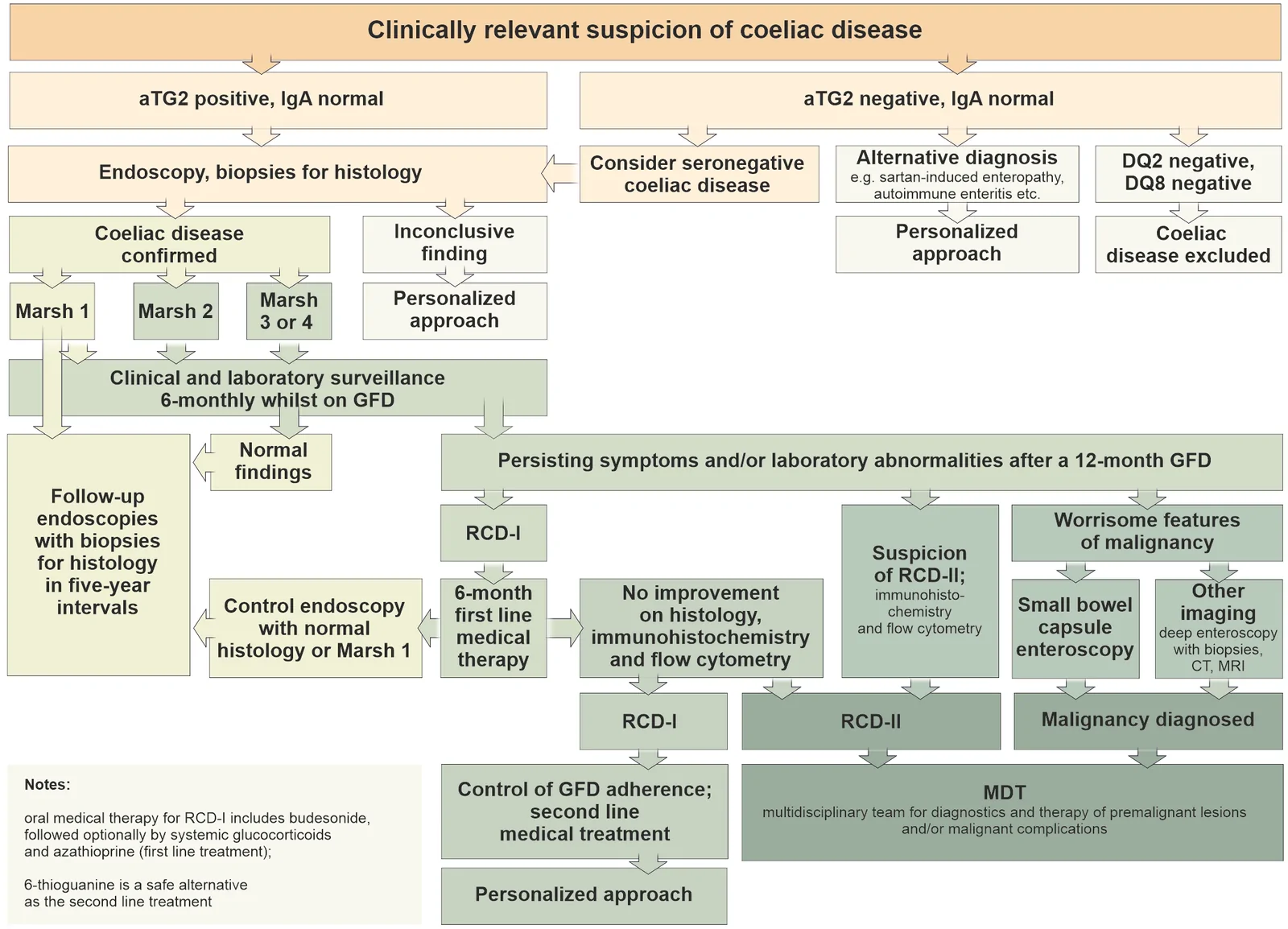
Simplified proposal of personalized surveillance of celiac disease and associated gastrointestinal premalignant conditions in adults. aTG2, serum type 2 anti-tissue transglutaminase IgA antibodies; IgA, serum concentration of immunoglobulin A; Marsh, Marsh-Rostami-Oberhuber classification; DQ2/DQ8, haplotypes of human leukocyte antigen (HLA) genes; GFD, strict gluten-free diet; RCD-I, type I refractory coeliac disease; RCD-II, type II refractory coeliac disease; CT, computed tomography; MDT, multidisciplinary team; MRI, magnetic resonance imaging.

## 12 Discussion

Current estimation of the prevalence of celiac disease is approximately 1% of the global population (89). These data are consistent with our current figures from the Czech Republic (prevalence of 1.06% in 2023). Celiac disease can be associated with several complications including a real risk of malignancy. Data concerning prevalence of refractory sprue are rather heterogeneous. Schiepatti et al. found persisting villous atrophy in 157/694 patients (23%; a 20-year cohort) (50). Other authors estimate the prevalence of refractory sprue to be substantially lower (<5%) (24). Enteropathy-associated T-cell lymphoma is more frequently diagnosed in adults from geographic areas with a high incidence of celiac disease, such as the western part of Ireland and Northern Europe (64). A large Dutch study found the incidence of enteropathy-associated T-cell lymphoma to be 0.1 per 100,000 subjects (69). Our 14-year data on a 10-million Czech population showed higher mean figures: an incidence of T-cell lymphoma of 0.44 per 100,000 for the general population, and an incidence of 1.91 per 100,000 for patients with T-cell lymphoma diagnosed with celiac disease.

We have proposed an algorithm for the surveillance of celiac disease with regard to premalignant lesions. There is a lack of universally accepted international recommendations in this aspect. We are aware of the controversial views on this topic, ranging widely from a proactive approach to skeptical reluctance. The first essential step is the correct diagnosis and differential diagnosis of celiac disease, particularly the seronegative form of the disease. In our algorithm, this relates to the section on negative serum type 2 anti-tissue transglutaminase IgA antibodies with normal serum concentration of immunoglobulin A. Seronegative celiac disease is associated with HLA-DQ2 and DQ8 positivity, occurring more frequently in elderly people with an autoimmune diathesis (90). Human leukocyte antigens (HLA class II) DQ2 and/or DQ8 are the most important genes predisposing individuals to celiac disease (91, 92). The HLA-DQ2 haplotype is the most frequently found (in approximately 90% of cases). Apart from celiac disease, DQ2 and DQ8 positivity is present in approximately 30% to 40% of the general population. The negative predictive value of the absence of both DQ2 and DQ8 is significant, as it almost certainly rules out celiac disease (93, 94).

In the differential diagnosis, drug-induced villous atrophy and autoimmune enteritis must always be considered (95–102). Sartan-induced enteropathy accounts for more than 80% of cases of drug-induced villous atrophy (96). Celiac disease serology is negative, the disease always carries autoimmune features, HLA-DQ2 is positive in 70%, and HLA-DQ8 is positive in 40% (97, 99). The autoimmune enteritis usually has a severe clinical course (e.g., vomiting and intractable diarrhea). A predisposition to other autoimmune reactions is characteristic. Diagnosis is based on the detection of circulating autoantibodies (GECA, AEA, and AGCA) (101, 102).

In patients with positive serum type 2 anti-tissue transglutaminase IgA antibodies and normal serum concentrations of immunoglobulin A, subsequent histology might be rarely inconclusive (e.g., collagen deposits and eosinophilic component). In such cases, a strictly personalized approach is mandatory. We found no exact guidance for follow-up endoscopy intervals. The follow-up endoscopy recommendation proposed in the algorithm represents an expert opinion based on our extensive clinical practice. The presented proposal will certainly be widely debated before its potential broader implementation into clinical practice.

A SWOT (Strengths, Weaknesses, Opportunities, and Threats) analysis (103, 104) provides a critical reflection of our project. Strengths offer and suggest a simple way to manage difficult cases of celiac disease. Graded controls are clearly defined and easy to be performed. Weaknesses consist in the fact that several opinion leaders do not consider endoscopy mandatory, not only for follow-up but even for initial diagnosis of celiac disease in adults. Electronic health records are not generally available. Major opportunities represent an option that results of follow-up can be easily implemented. Premalignant lesions can be treated effectively; malignant complications can be diagnosed at an earlier stage and thus the treatment can be started without any purposeless delay. Threats are mostly represented by the different availability and uneven quality of healthcare worldwide (41).

The strength of this paper is that it provides exact epidemiological data on celiac disease, T-cell lymphoma, and small intestinal adenocarcinoma in the Czech Republic over the period 2010–2023. Another contribution is our proposed priority surveillance algorithm for adults with celiac disease with regard to premalignant lesions. However, we are aware of its possible limits. The algorithm does not, and cannot, cover all clinical situations that may arise. Another limitation is the fact that the algorithm will probably not be possible to validate. Nevertheless, it offers new concepts of personalized approach to a patient with adult celiac disease and new directions of future research.

## 13 Conclusions

Graded follow-up of adult celiac disease has become mandatory. Although the risk of malignancy is relatively low, especially in a strict gluten-free diet, it might be further decreased by an individualized surveillance. Endoscopy controls with biopsies for histology, immunohistochemistry, and flow cytometry are essential. A proposal for the individualized surveillance of premalignant gastrointestinal lesions in adult celiac disease was drafted.
