# Supplementary material for: Risk of malignancy and surveillance of premalignant gastrointestinal lesions in adult celiac disease
Source: Front Oncol. 2026 Sep 3;16:1876345. doi: 10.3389/fonc.2026.1876345 (PMC13583916; doi:10.3389/fonc.2026.1876345)

Supplementary Materials

Figure 1
Normal duodenum. White-light endoscopy shows normal appearance of the duodenum, with dense transverse folds and high villi.

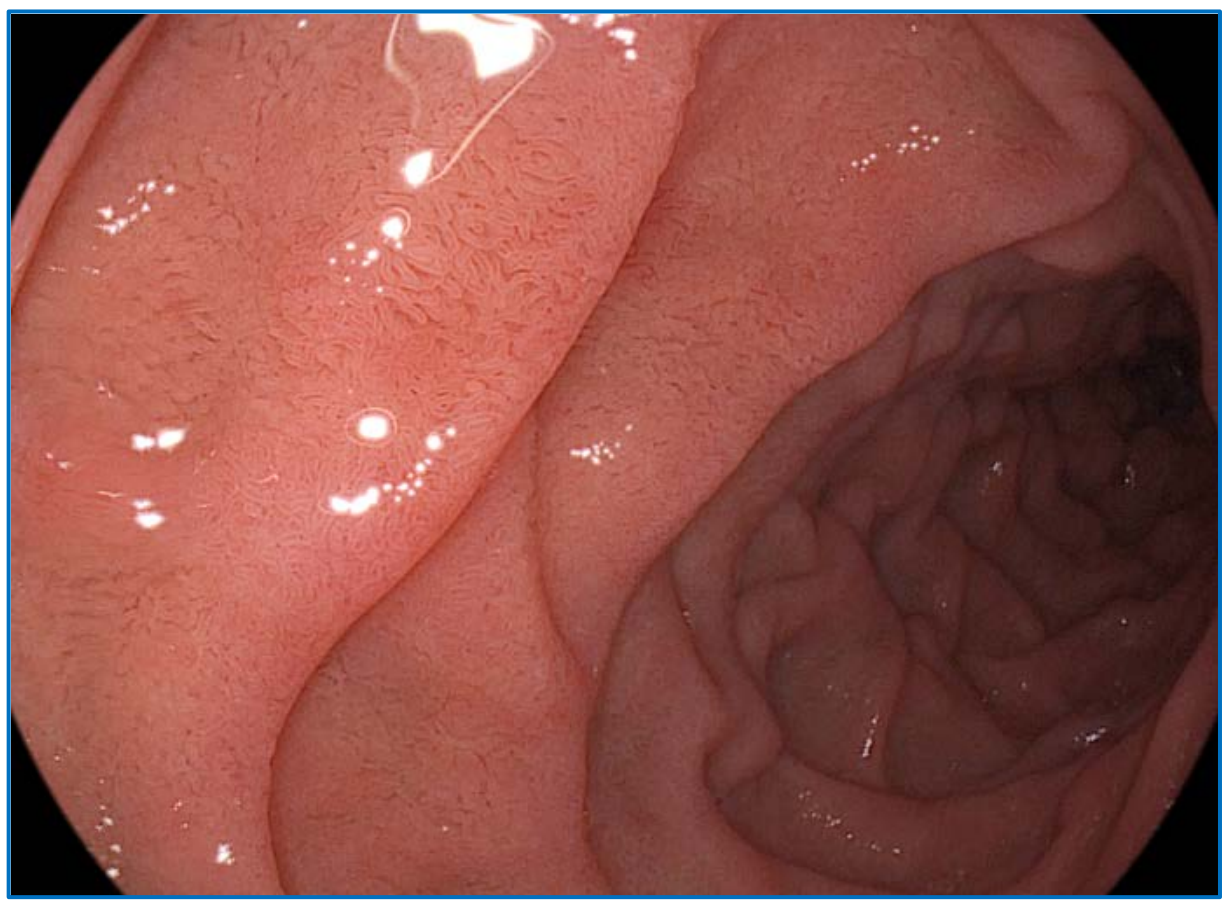

Figure 2
Normal small intestine. Small bowel mucosa with normal villi (yellow bracket and arrow) and crypts (blue bracket and arrow) in 4:1 ratio, intraepithelial lymphocytes scattered, in normal range (< 25 for 100 epithelial cells). *Hematoxylin-eosin, original magnification 100x.*

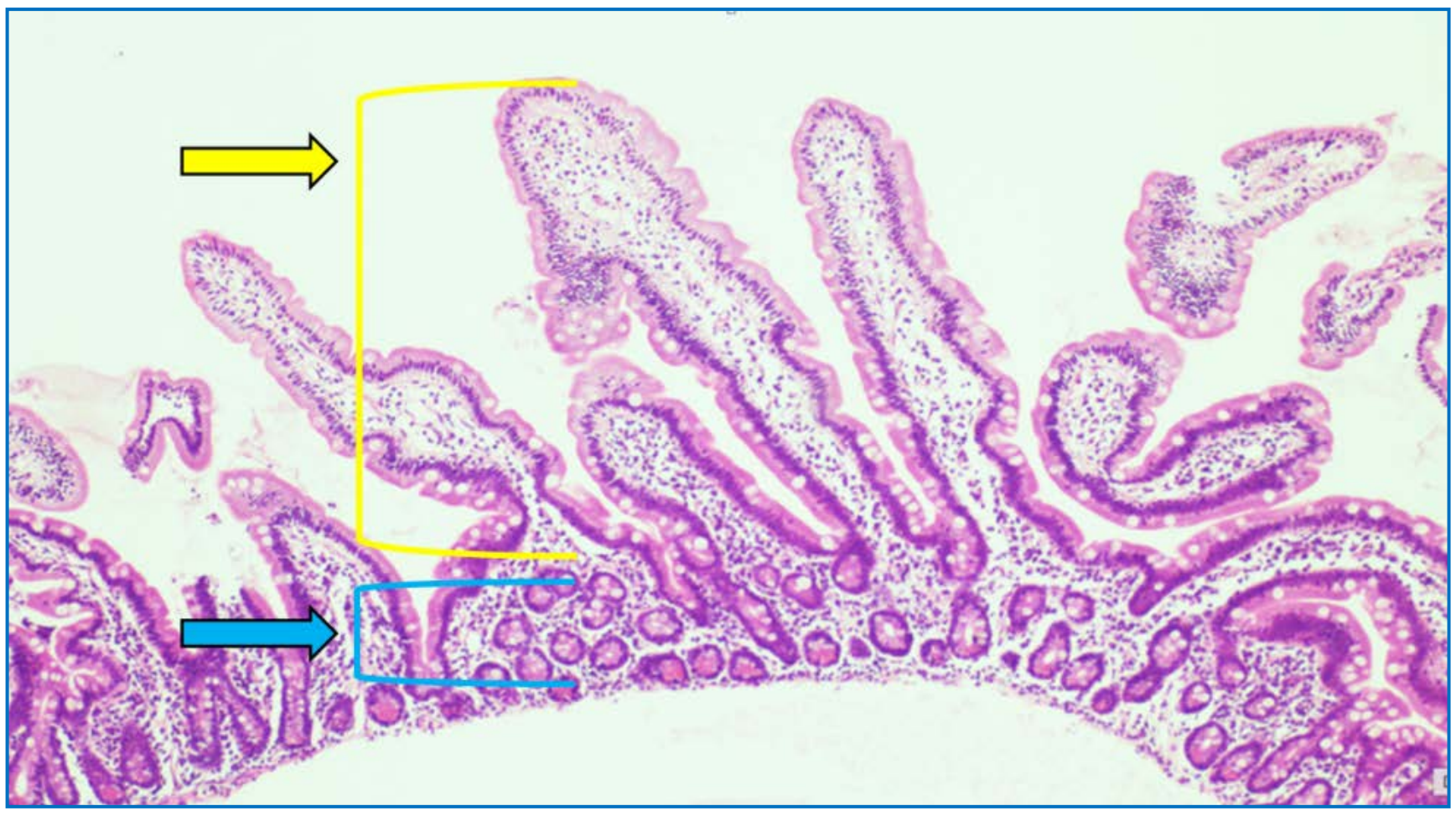

Figure 3
Celiac disease. White-light endoscopy of the proximal jejunum, with dilated lumen, reduced transverse folds and absence of villi. The most characteristic endoscopic feature of celiac disease is a mosaic pattern. Reproduced with permission from Bures et al. [ref. 35].

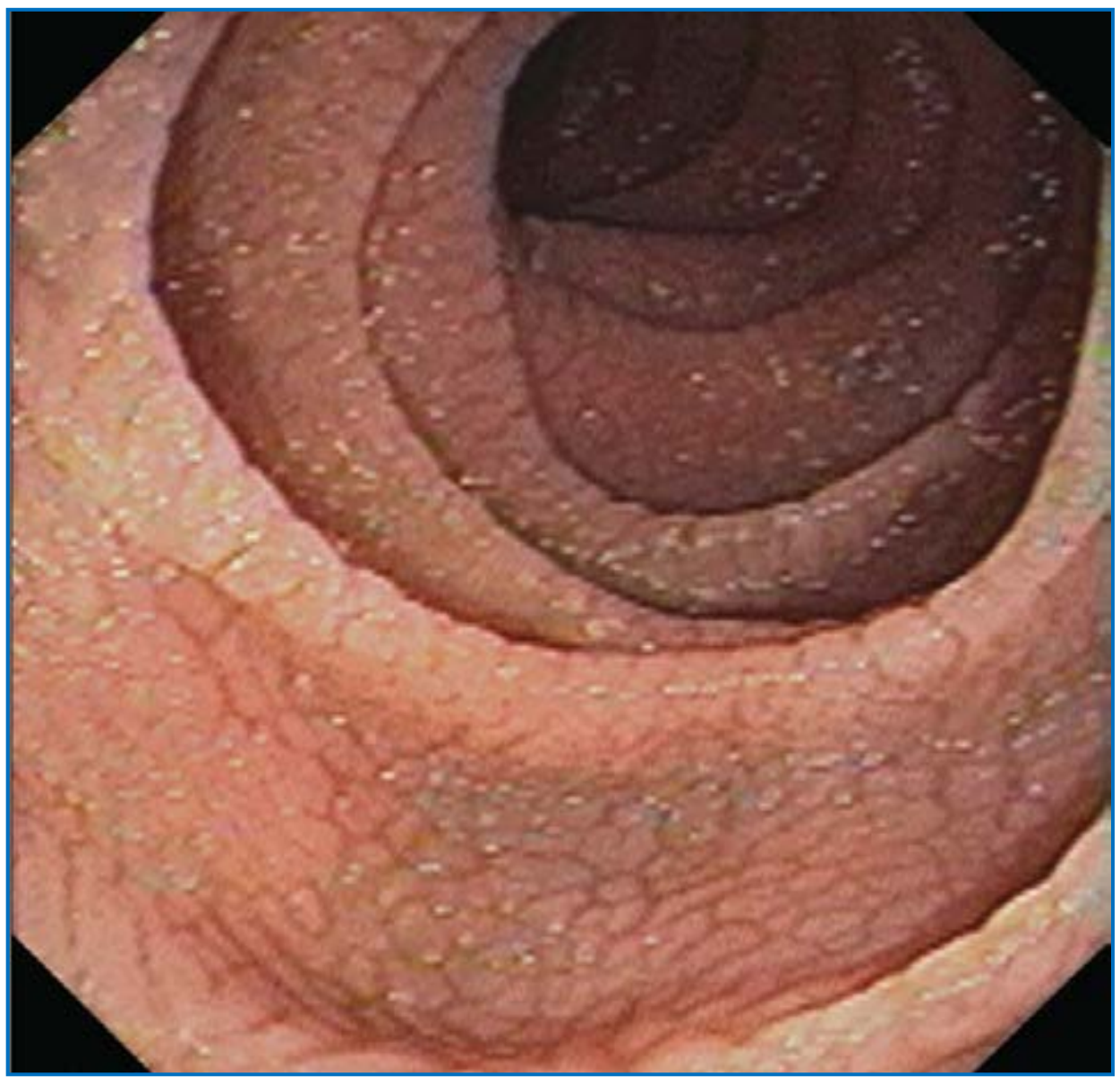

Figure 4
Celiac disease. Small bowel mucosa with almost complete atrophy of villi (yellow bracket and arrow) with increased intraepithelial lymphocytosis and crypt hyperplasia (blue bracket and arrow), corresponding with diagnosis of celiac disease Marsh 3c (Marsh-Rostami-Oberhuber classification). *Hematoxylin-eosin, original magnification 200x.*

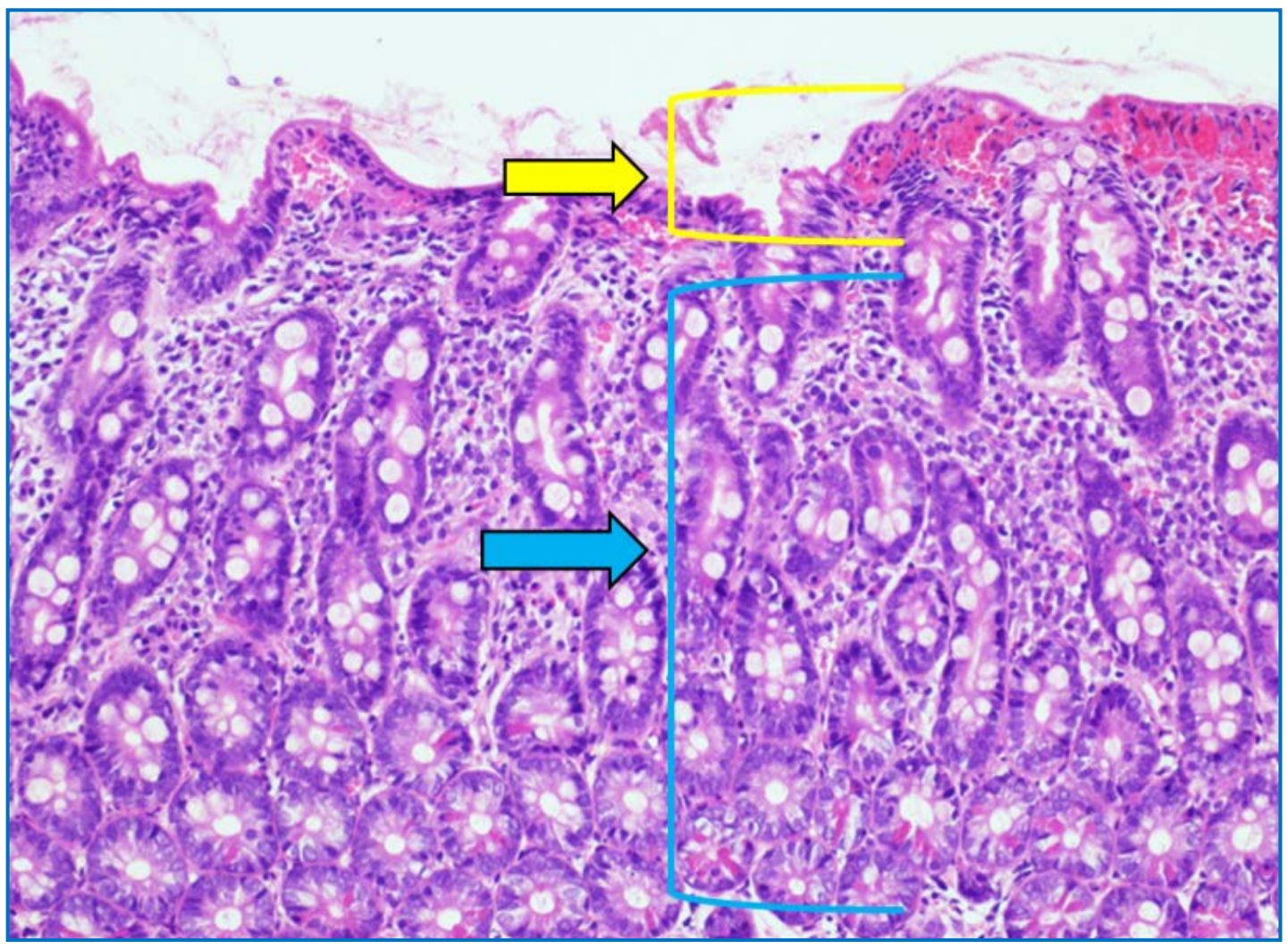

Figure 5
Celiac disease. CD3 immunohistochemistry highlighting intraepithelial lymphocytes (green arrows) in range up to 60 per 100 enterocytes. *Original magnification 200x.*

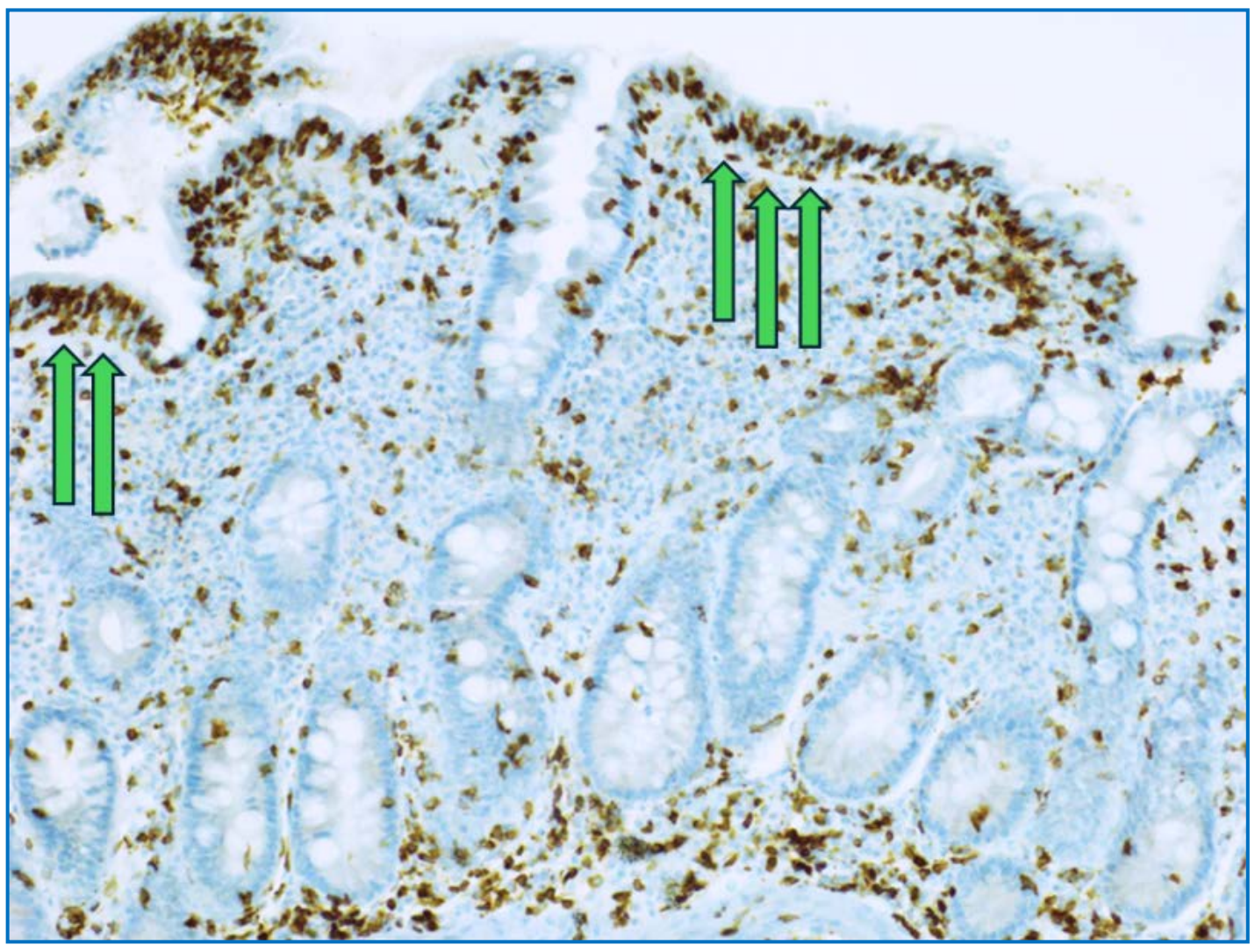

Figure 6
Type I refractory celiac disease. White-light endoscopy. Severe involvement of the proximal jejunum. Swelling in atrophic mucosa creates irregular nodular pattern of the jejunum. Transverse folds are absent, mucosa is reddish in colour. Reproduced with permission from Bures et al. [ref. 35].

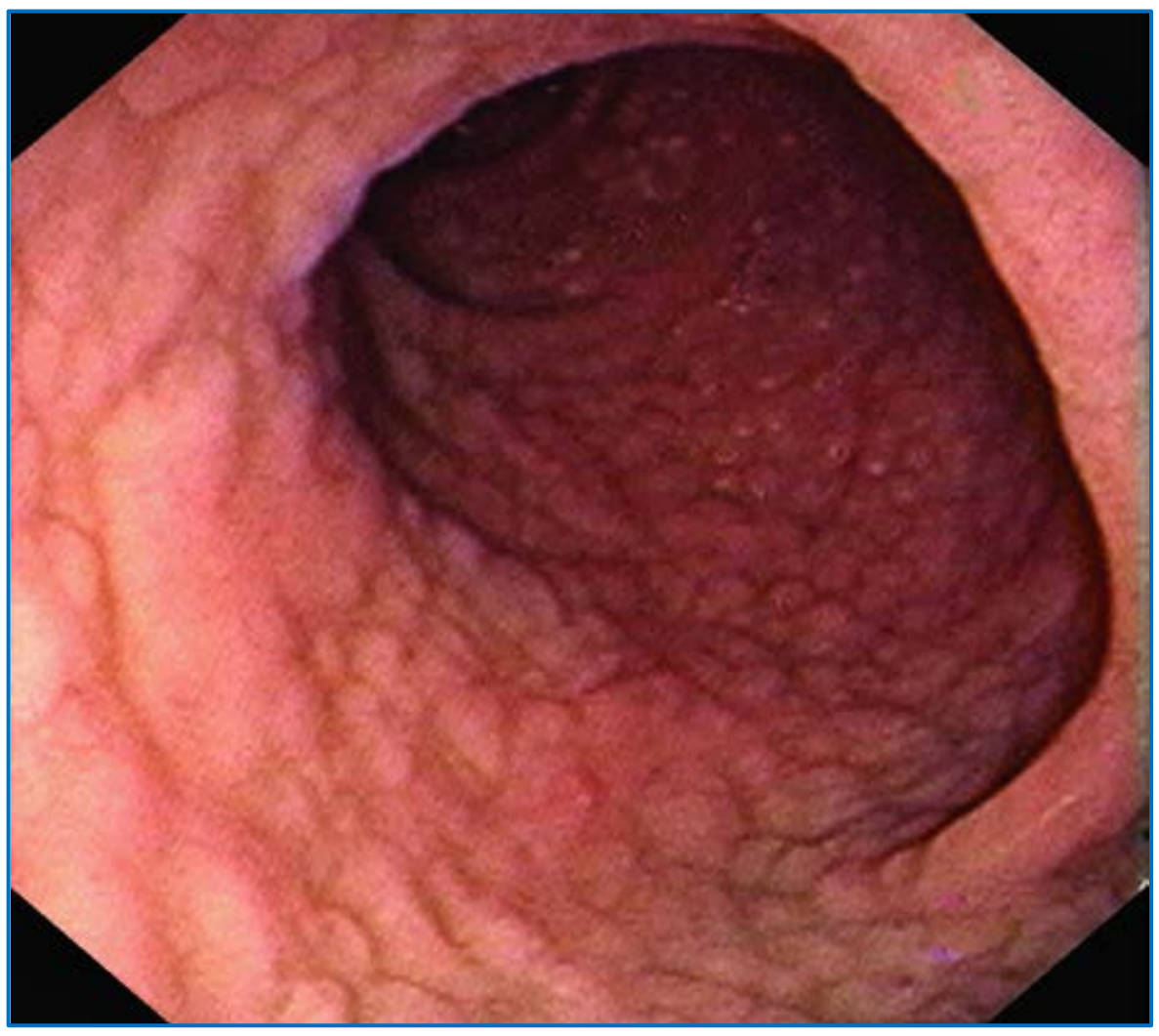

Figure 7
Type I refractory celiac disease with partial villous atrophy and persistent intraepithelial lymphocytosis without aberrant phenotype. CD8 expression is reduced by only 23% compared to CD3. Small bowel mucosa with slightly shortened villi (yellow bracket and arrow), intraepithelial lymphocytosis and mild crypt hyperplasia (blue bracket and arrow) corresponding with celiac disease Marsh 3a (Marsh-Rostami-Oberhuber classification). *Hematoxylin-eosin, original magnification 100x.*

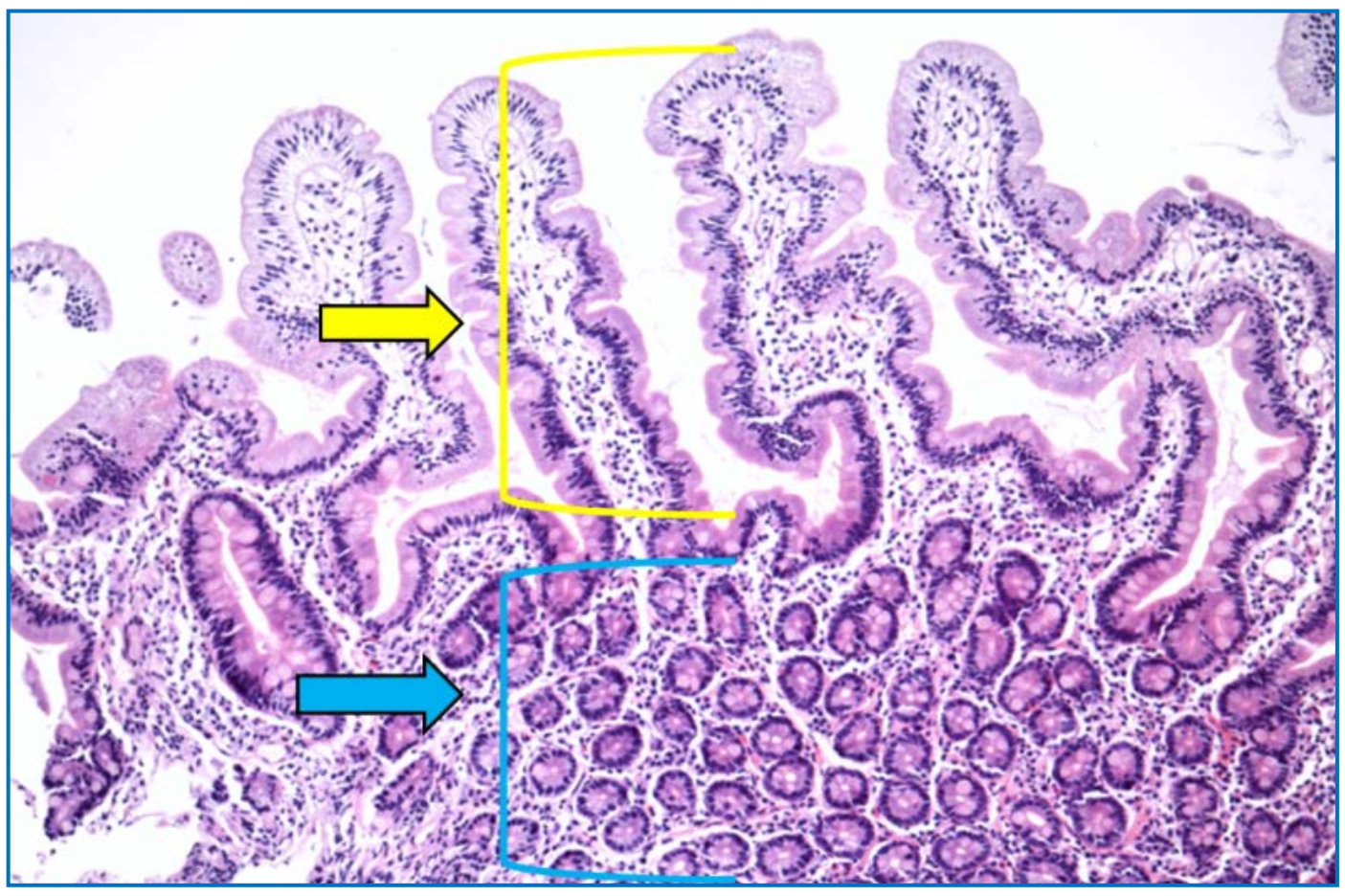

Figure 8
Type I refractory celiac disease. CD3 immunohistochemistry highlighting intraepithelial lymphocytes in range up to 45 per 100 enterocytes (orange arrows). *Original magnification 200x.*

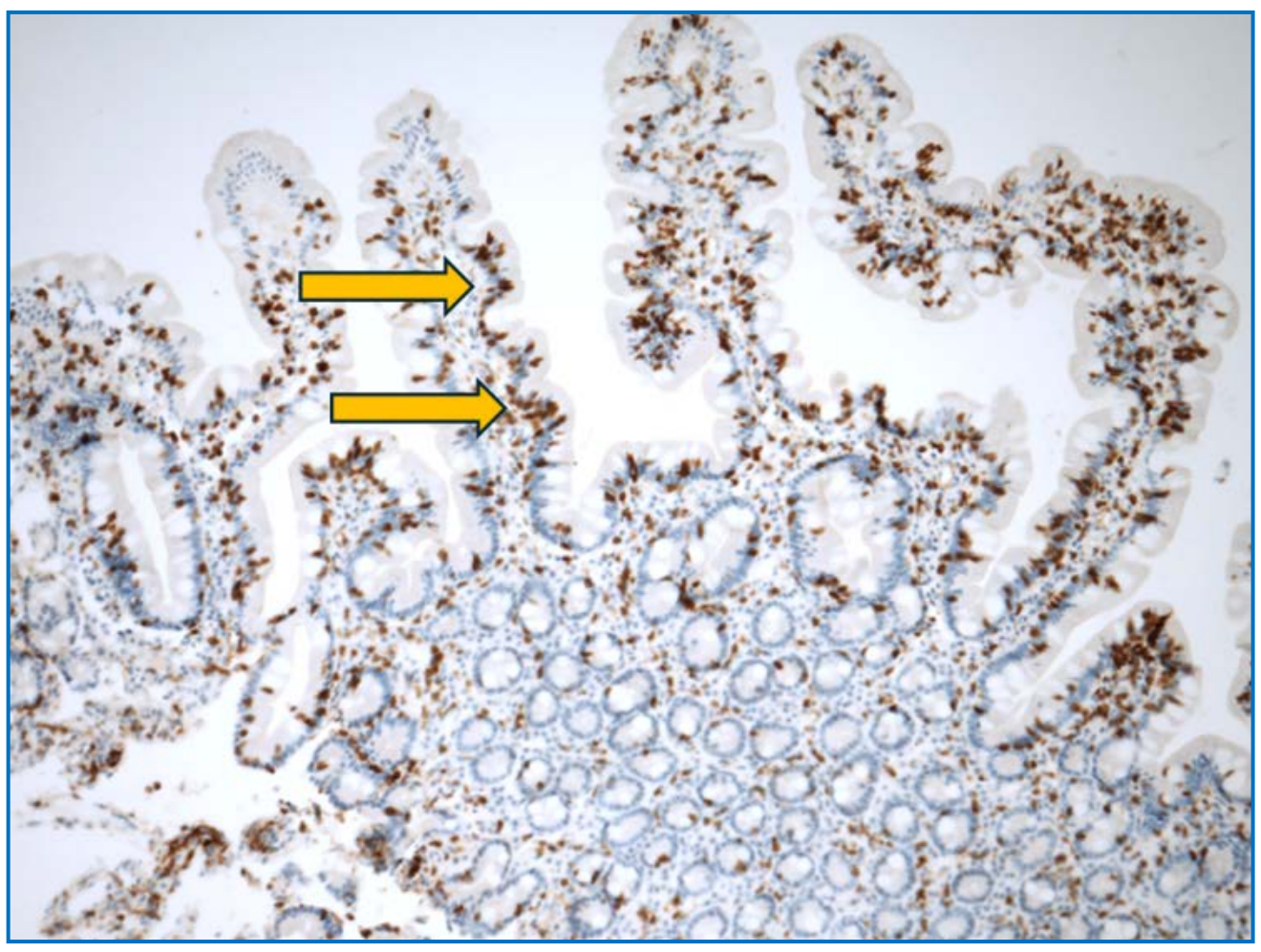

Figure 9
Type I refractory celiac disease. CD8 immunohistochemistry highlighting intraepithelial lymphocytes in range up to 35 per 100 enterocytes (purple arrows). *Original magnification 200x.*

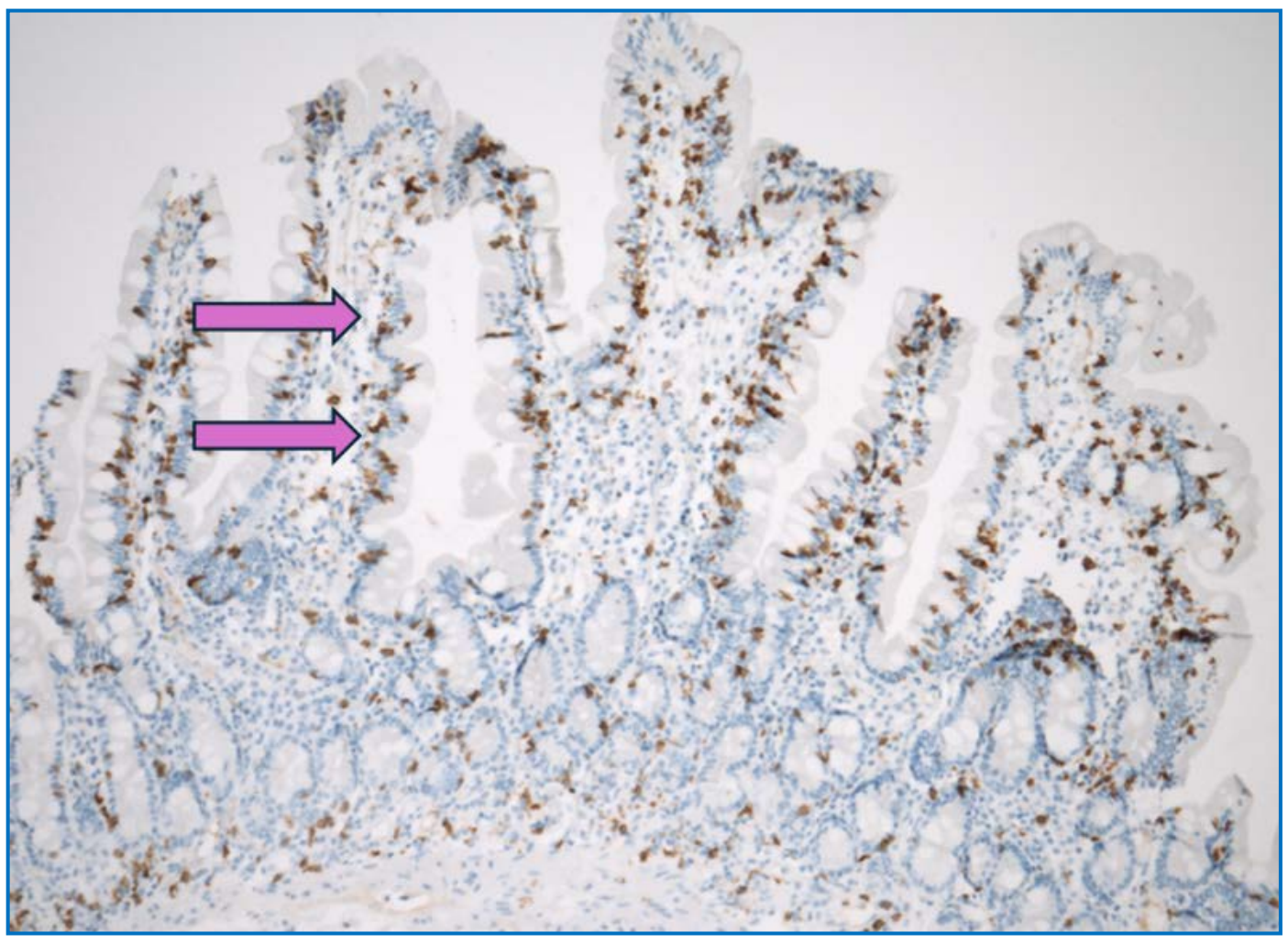

Figure 10
Type II refractory celiac disease. White-light endoscopy. Stenosis of the D3 duodenum and two ulcers (arrows) in front of stenosis. Reproduced with permission from Bures et al. [ref. 35].

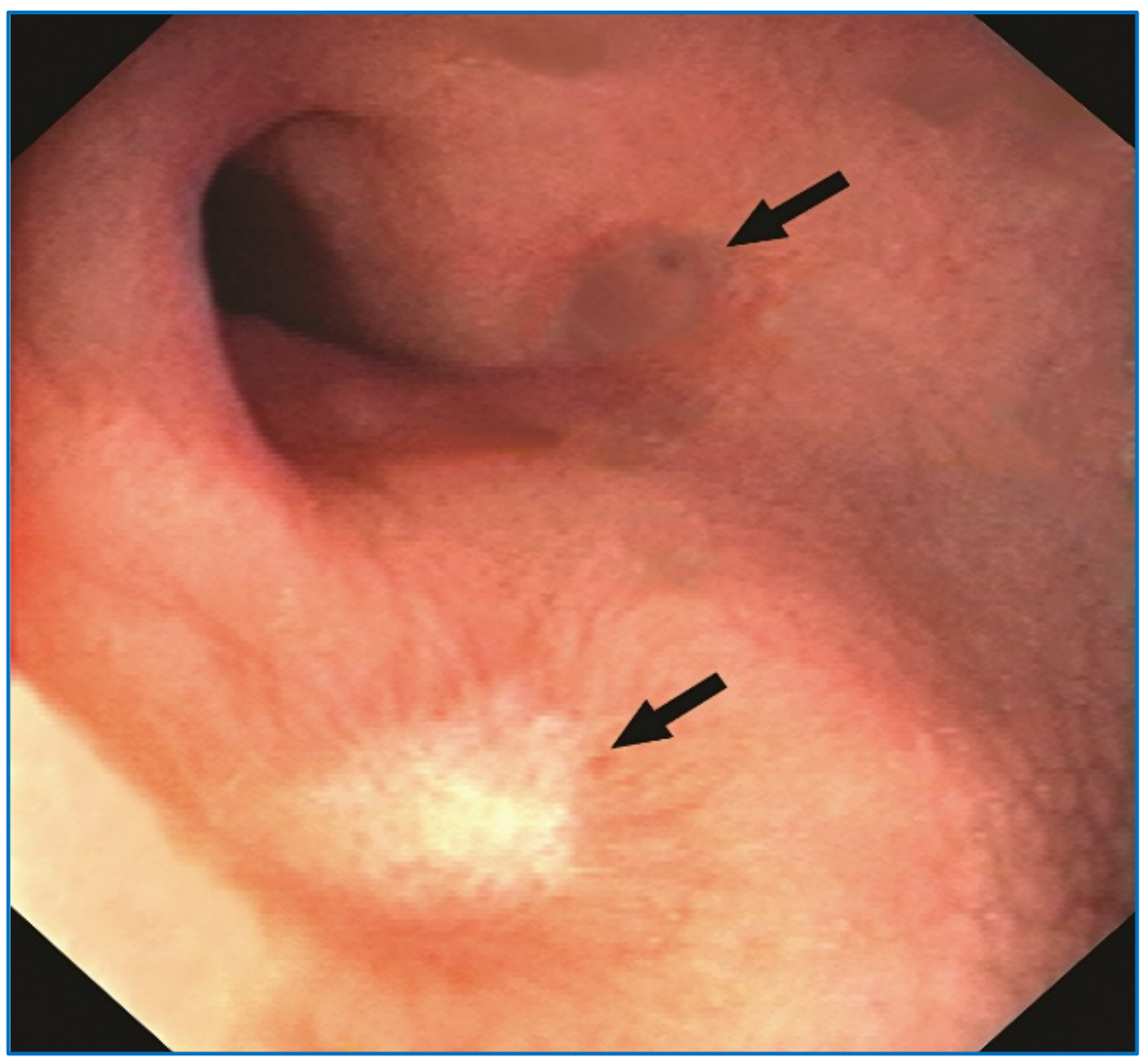

Figure 11
Type II refractory celiac disease with almost complete villous atrophy and persistent intraepithelial lymphocytosis with aberrant phenotype. CD8 expression is reduced by 70% compared to CD3. Flattened small bowel mucosa with intraepithelial lymphocytosis (green arrows) and prominent crypt hyperplasia corresponding with celiac disease Marsh 3b/c (Marsh-Rostami-Oberhuber classification). *Hematoxylin-eosin, original magnification 100x.*

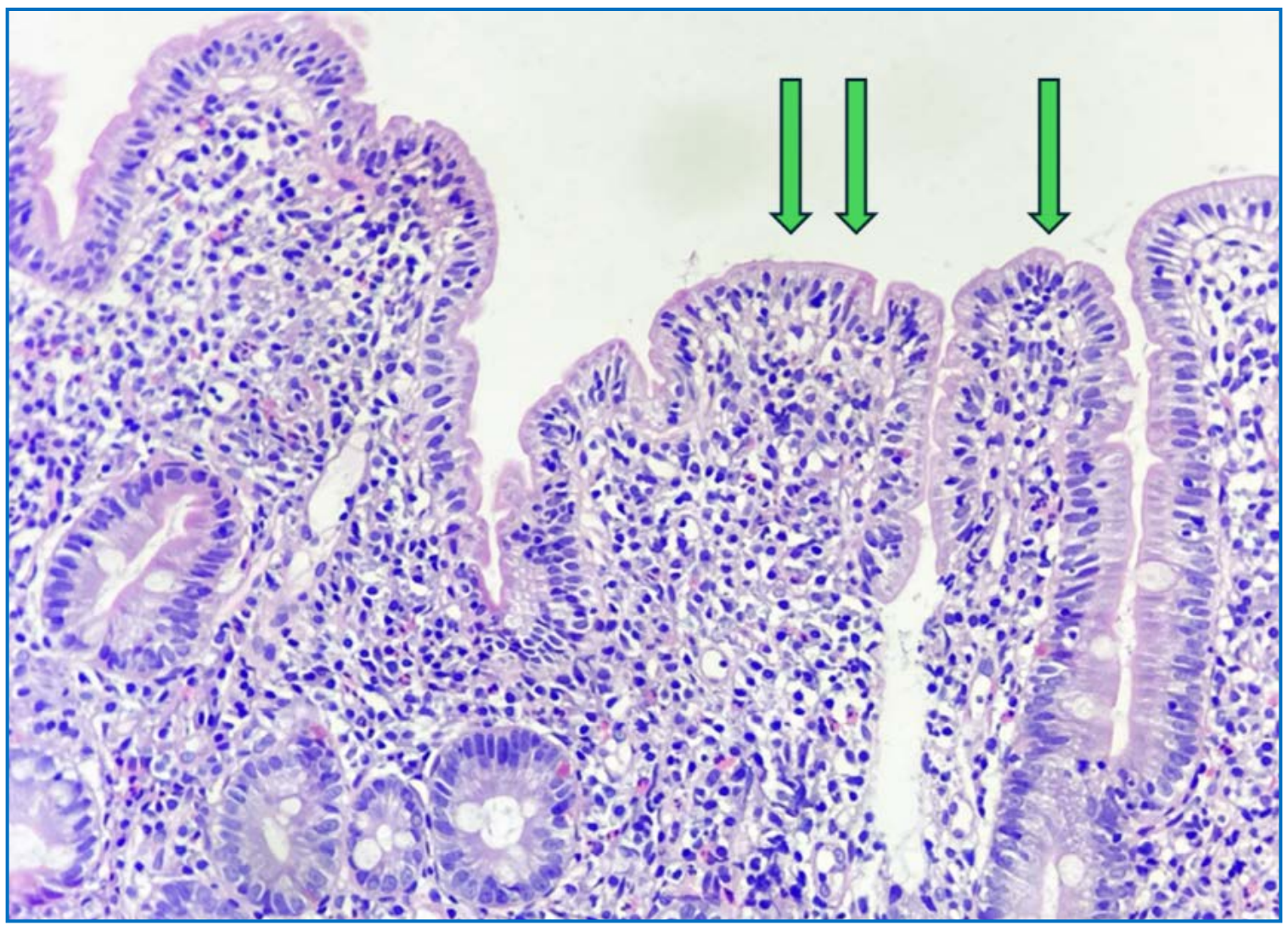

Figure 12
Type II refractory celiac disease. CD3 immunohistochemistry highlighting intraepithelial lymphocytes in range up to 50 per 100 enterocytes (orange arrows). *Original magnification 200x.*

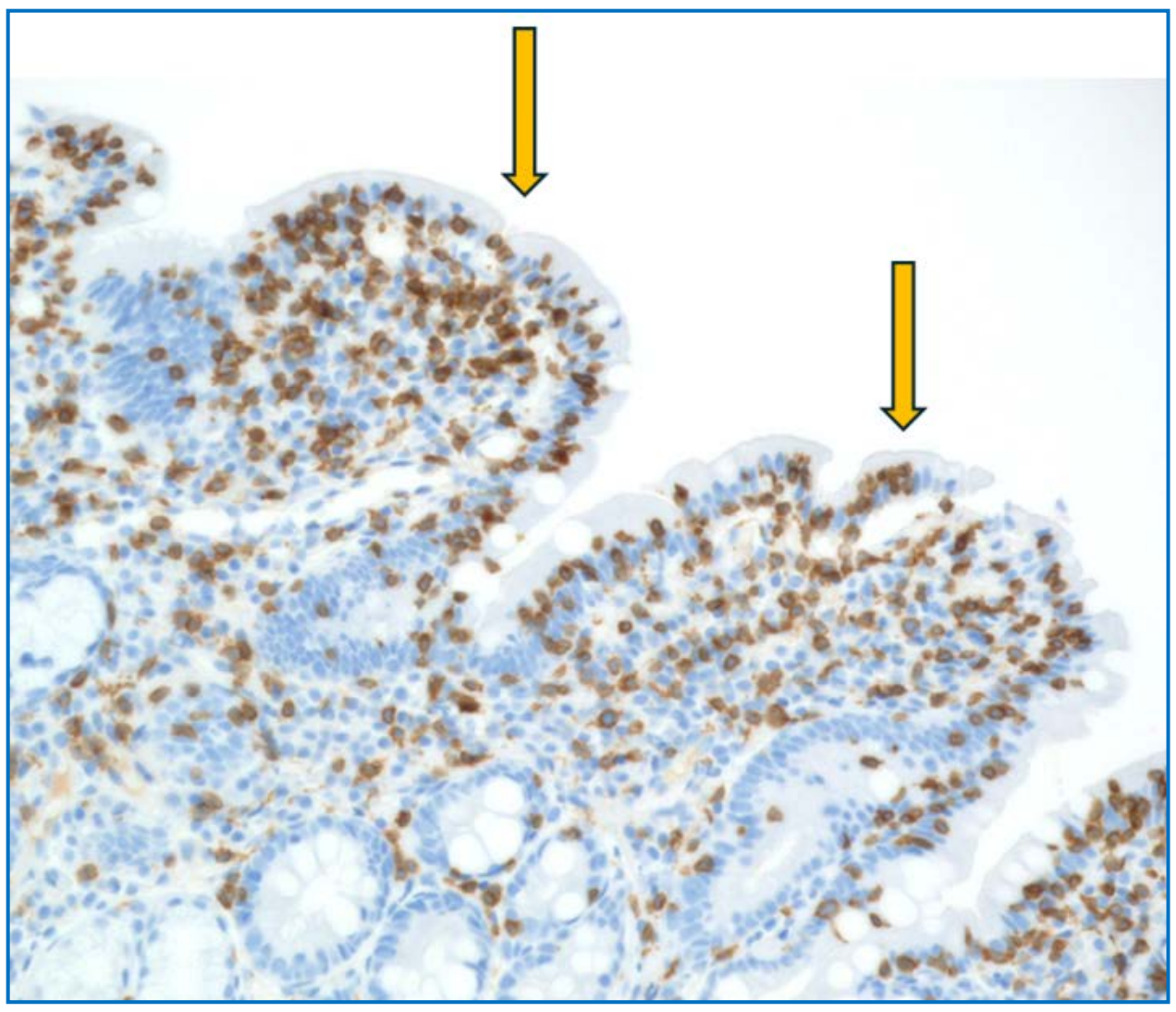

Figure 13
Type II refractory celiac disease. CD8 immunohistochemistry highlighting intraepithelial lymphocytes in range up to 15 per 100 enterocytes (purple arrows). *Original magnification 200x.*

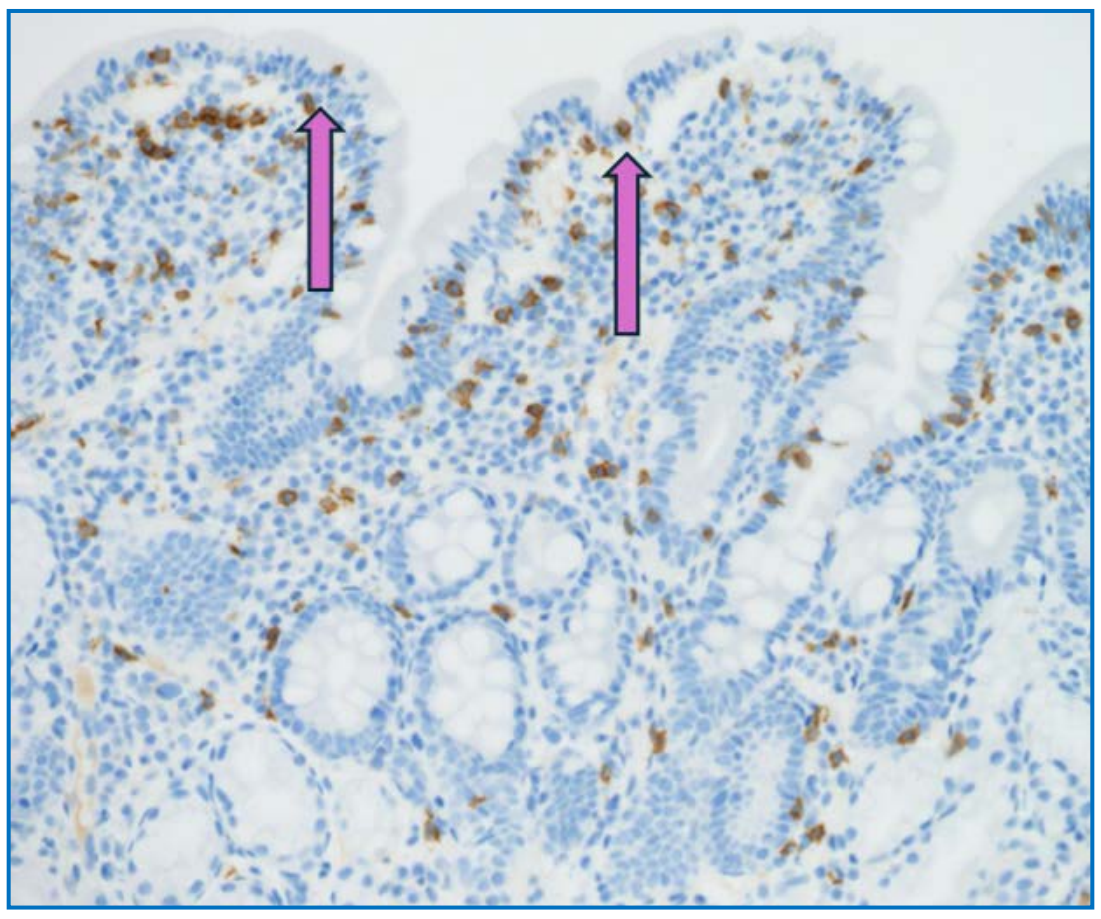

Figure 14
Flow cytometry of a small intestinal biopsy specimen. On the left: gating lymphocytes (process of selecting a subset of lymphocytes) using a surface expression of the CD45 marker. On the right: gating intraepithelial lymphocytes using surface expression of the CD103 marker.

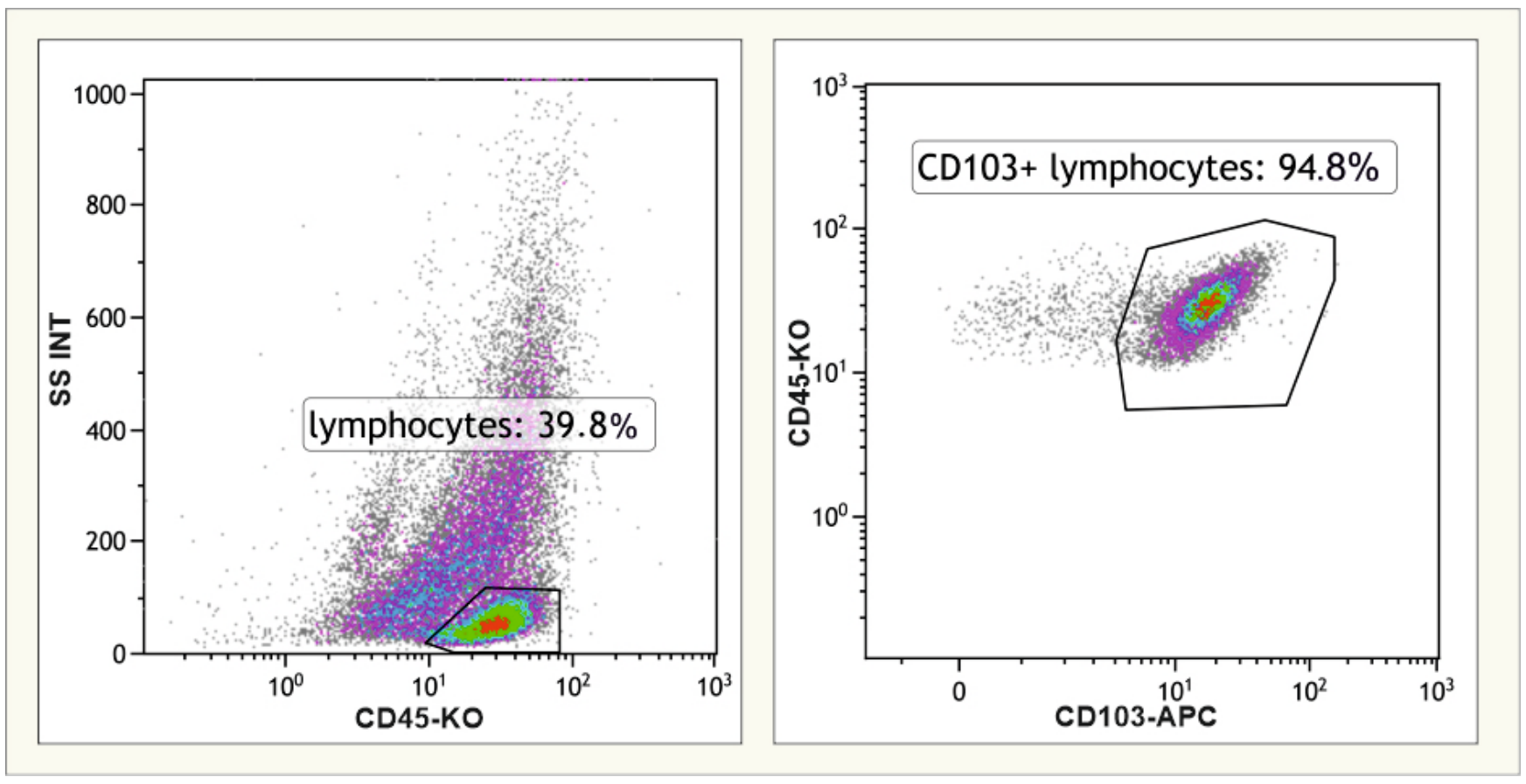

Figure 15
Flow cytometry in type II refractory celiac disease. Small intestinal biopsy specimen. On the left: classification (clustering) of T lymphocytes in dependence on surface expression of sCD3 and cytoplasmic expression of cCD3 into normal T lymphocytes (blue colour) and abnormal T lymphocytes with loss of surface expression of CD3 (red colour). In the middle and on the right: abnormal T lymphocytes which neither express other T lymphocyte markers such as CD5, CD8 or TCR gamma delta receptor, nor the NK cell marker CD56.

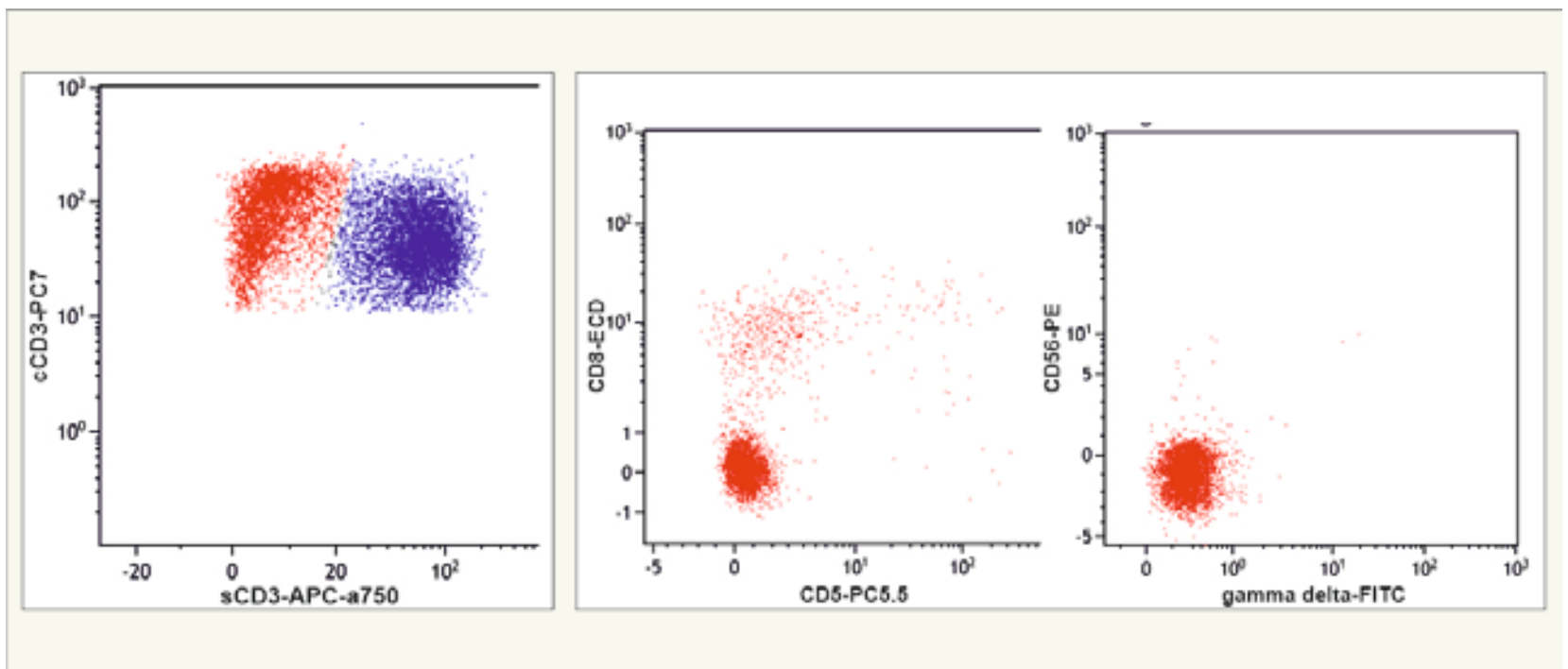

Figure 16
Enteropathy-associated T cell lymphoma in celiac disease. White-light endoscopy. Affected part of the distal duodenum is rigid (open arrows), infiltrated mucosa is fragile and reveals granular pattern. Reproduced with permission from Bures et al. [ref. 35].

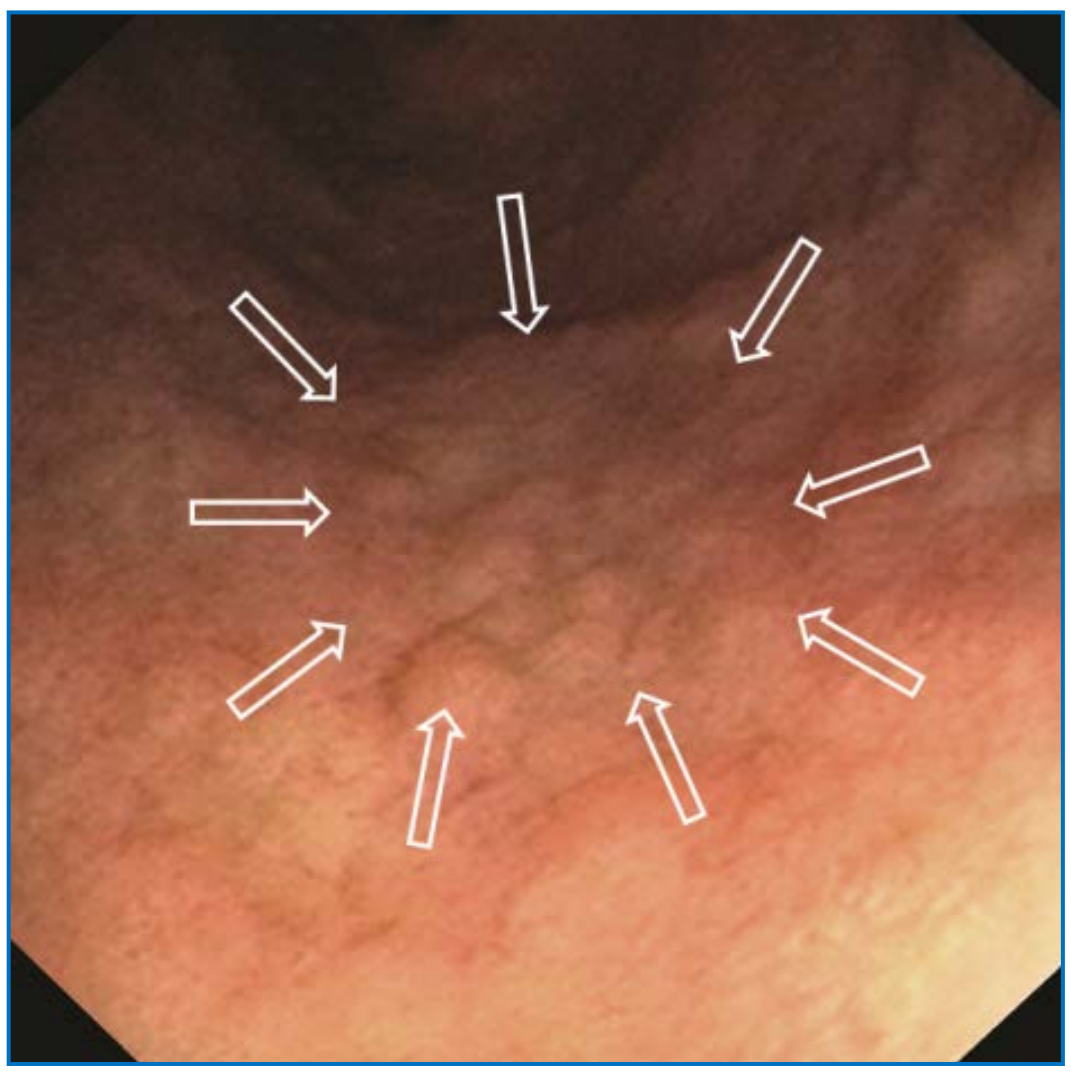

Figure 17
Enteropathy-associated T cell lymphoma in celiac disease. A section of the small intestine surgical specimen in overview magnification with lymphoma infiltration affecting the mucosal connective tissue of the expanded villi and part of the submucosa. *Hematoxylin-eosin, original magnification 40x.*

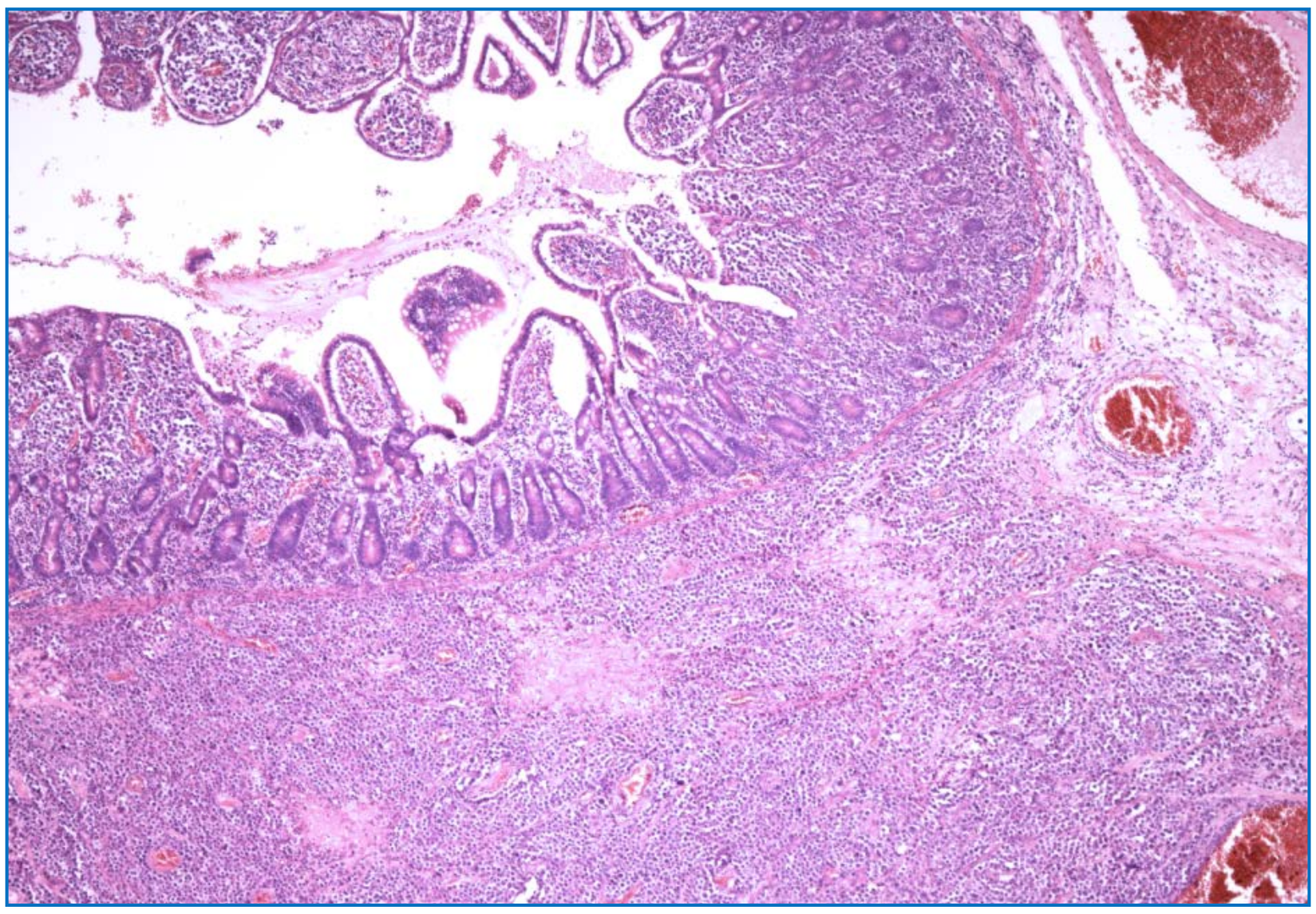

Figure 18
Enteropathy-associated T cell lymphoma in celiac disease. Mucosa and submucosa with infiltration by T lymphocytes in shortened villi, including intraepithelial infiltration (green arrows). *Hematoxylin-eosin, original magnification 100x.*

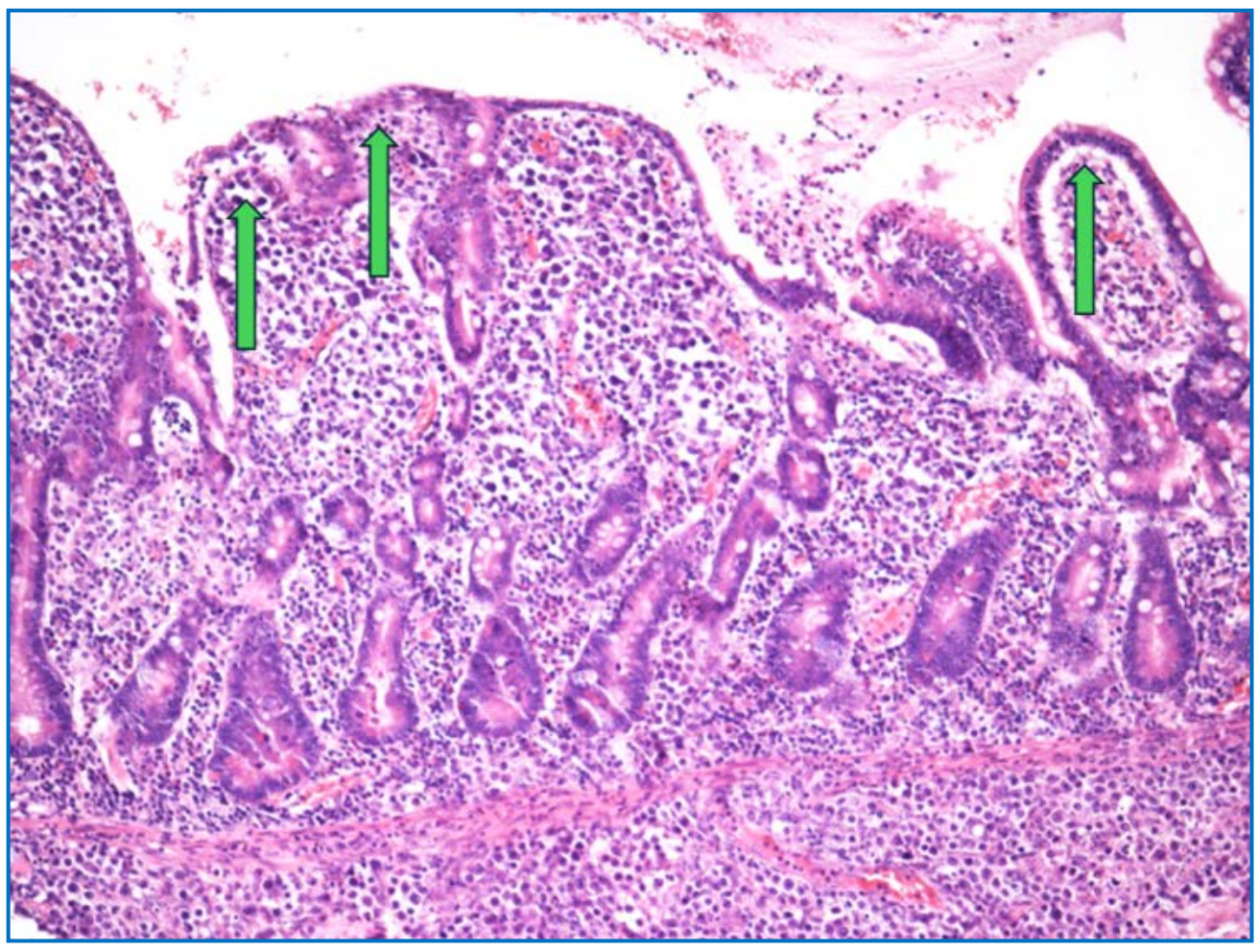

Figure 19
Enteropathy-associated T cell lymphoma in celiac disease. CD3 immunohistochemistry highlighting tumorous and intraepithelial lymphocytes (orange arrows) with predominantly aberrant cytoplasmic positivity. *Original magnification 100x.*

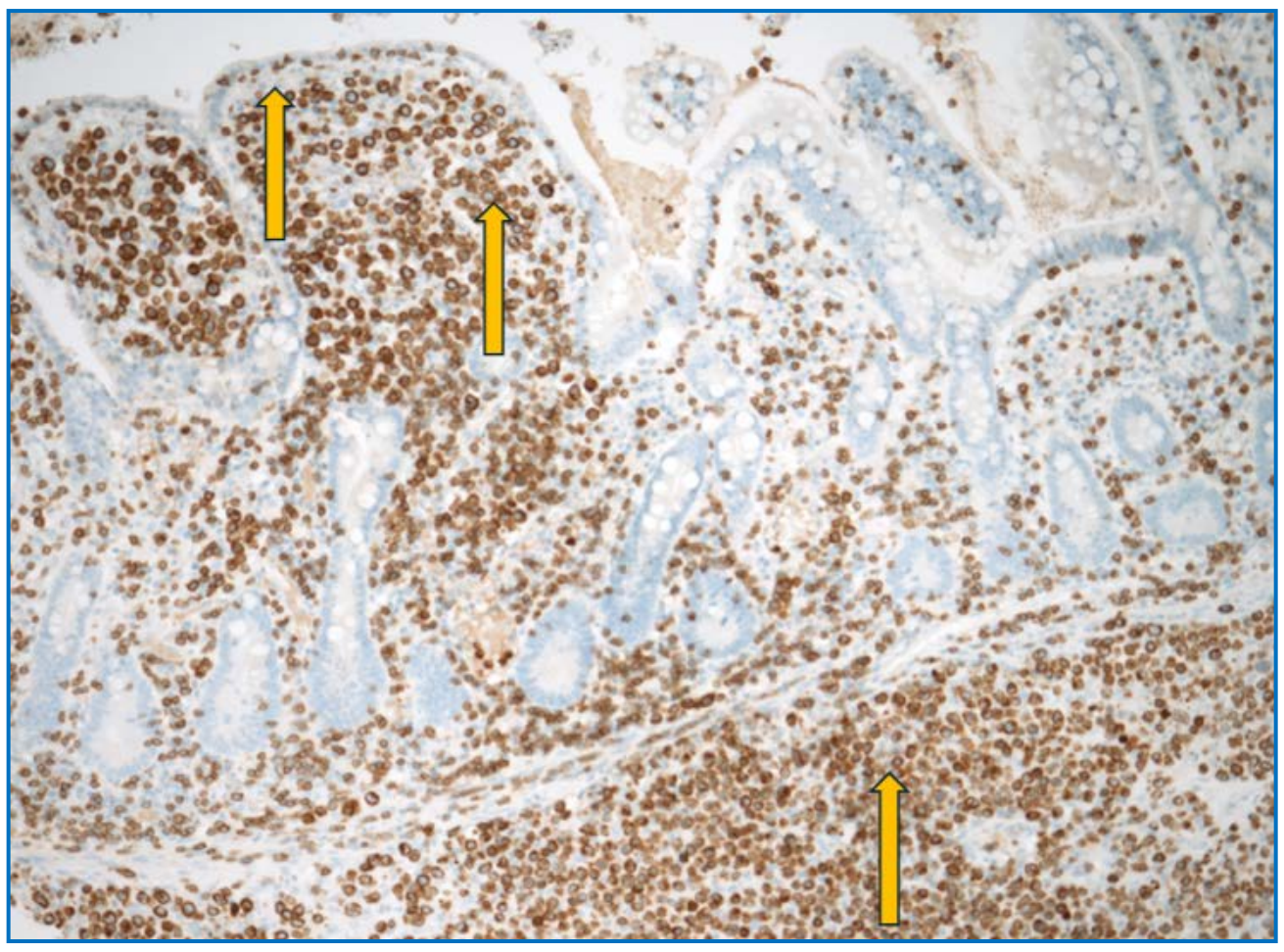

Figure 20
Diffuse large B cell lymphoma of the terminal ileum and caecum. Fluorine-18 Fluorodeoxyglucose (18F-FDG) Positron Emission Tomography/Computed Tomography (PET/CT), fusion image, transverse section. Large mass of lymphoma with intense 18F-FDG uptake was found (arrows).

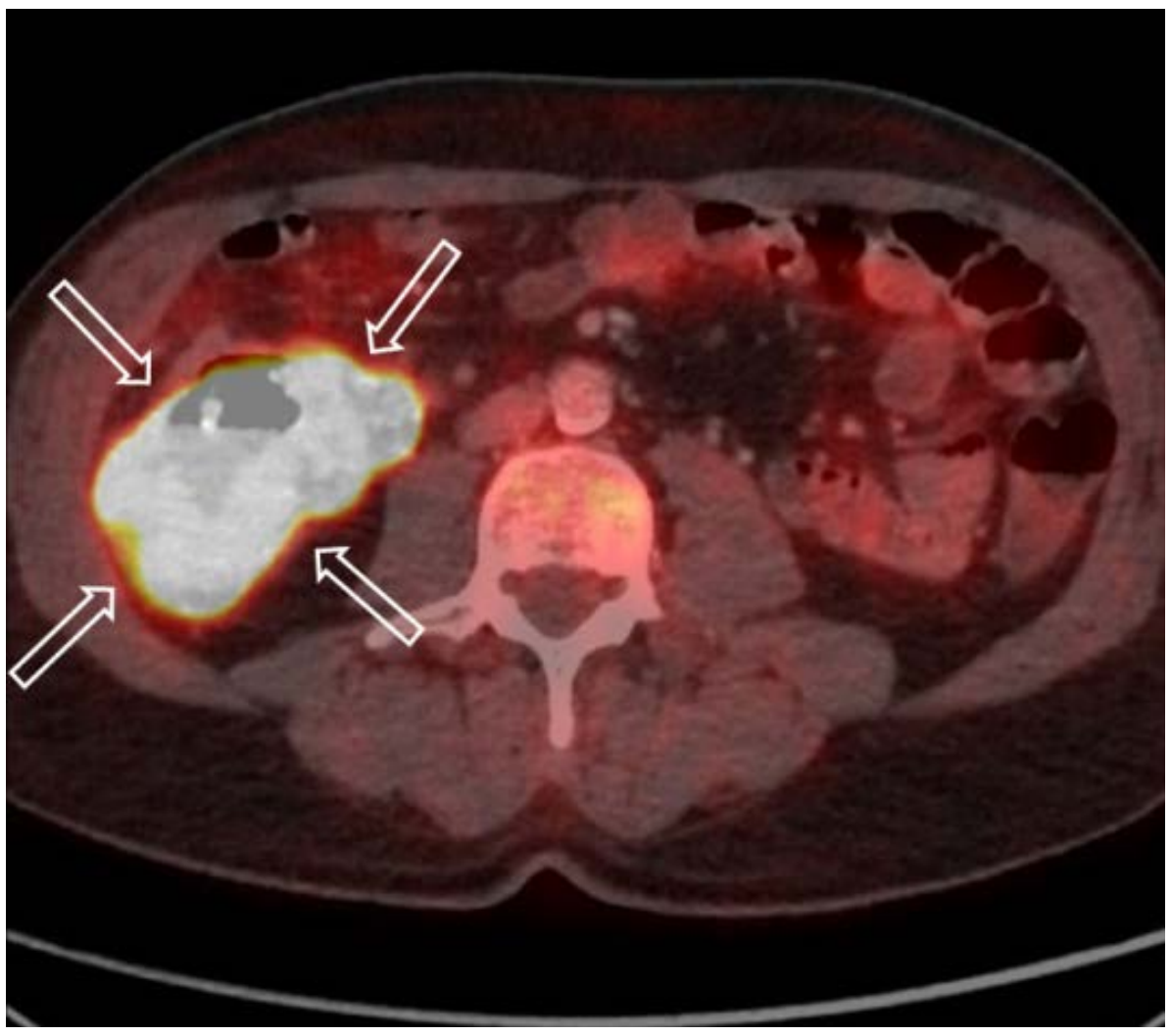

Figure 21
Folicular B cell lymphoma of the terminal ileum. Fluorine-18 Fluorodeoxyglucose (18F-FDG) Positron Emission Tomography/Computed Tomography (PET/CT), fusion image, coronal section. Intense 18F-FDG uptake in the terminal ileum is marked with an arrow.

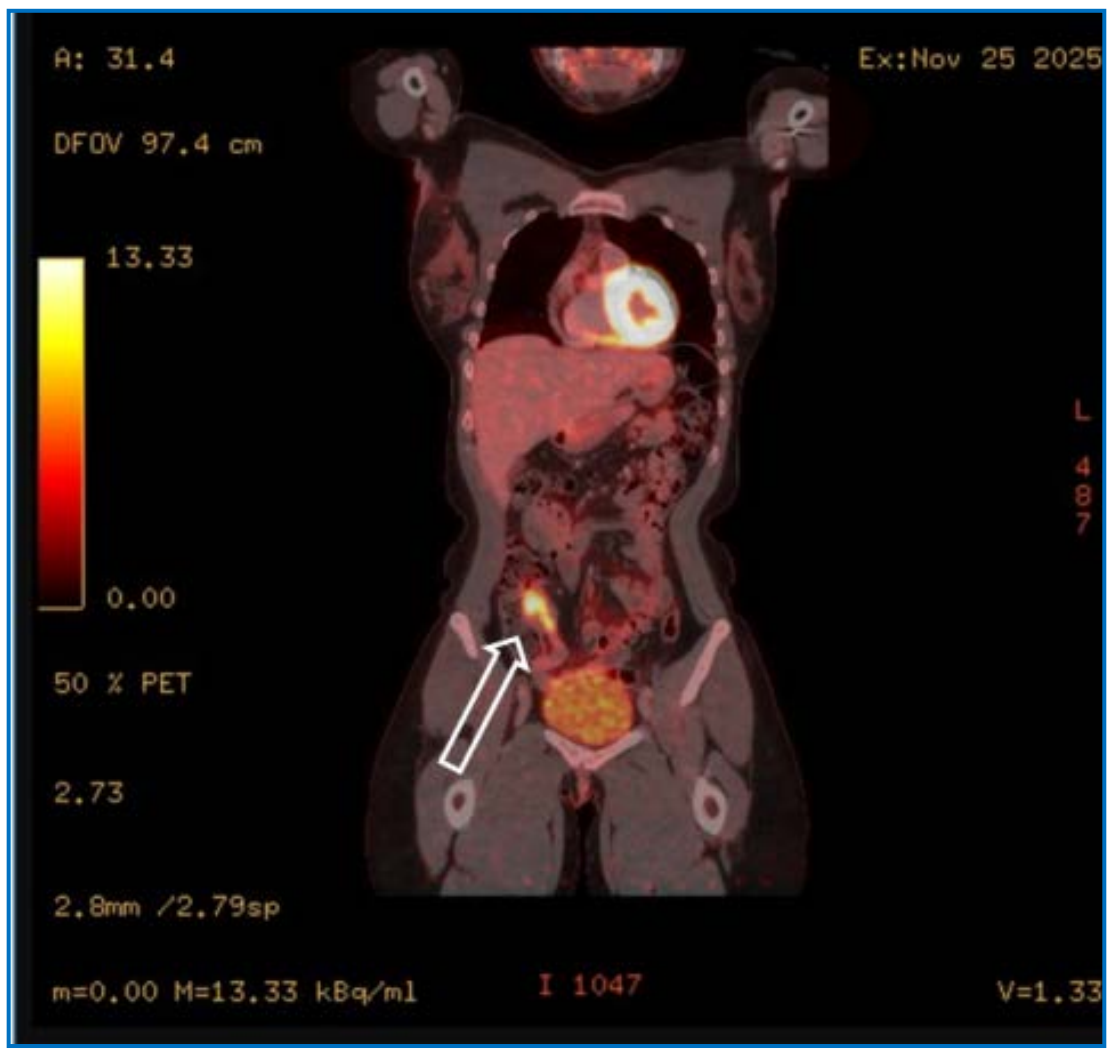

Figure 22
Adenocarcinoma of the proximal jejunum. Wireless small bowel capsule endoscopy. Stenosing tumour with oozing bleeding (open arrow), dilated lymphatic vessels on the left (closed arrows).

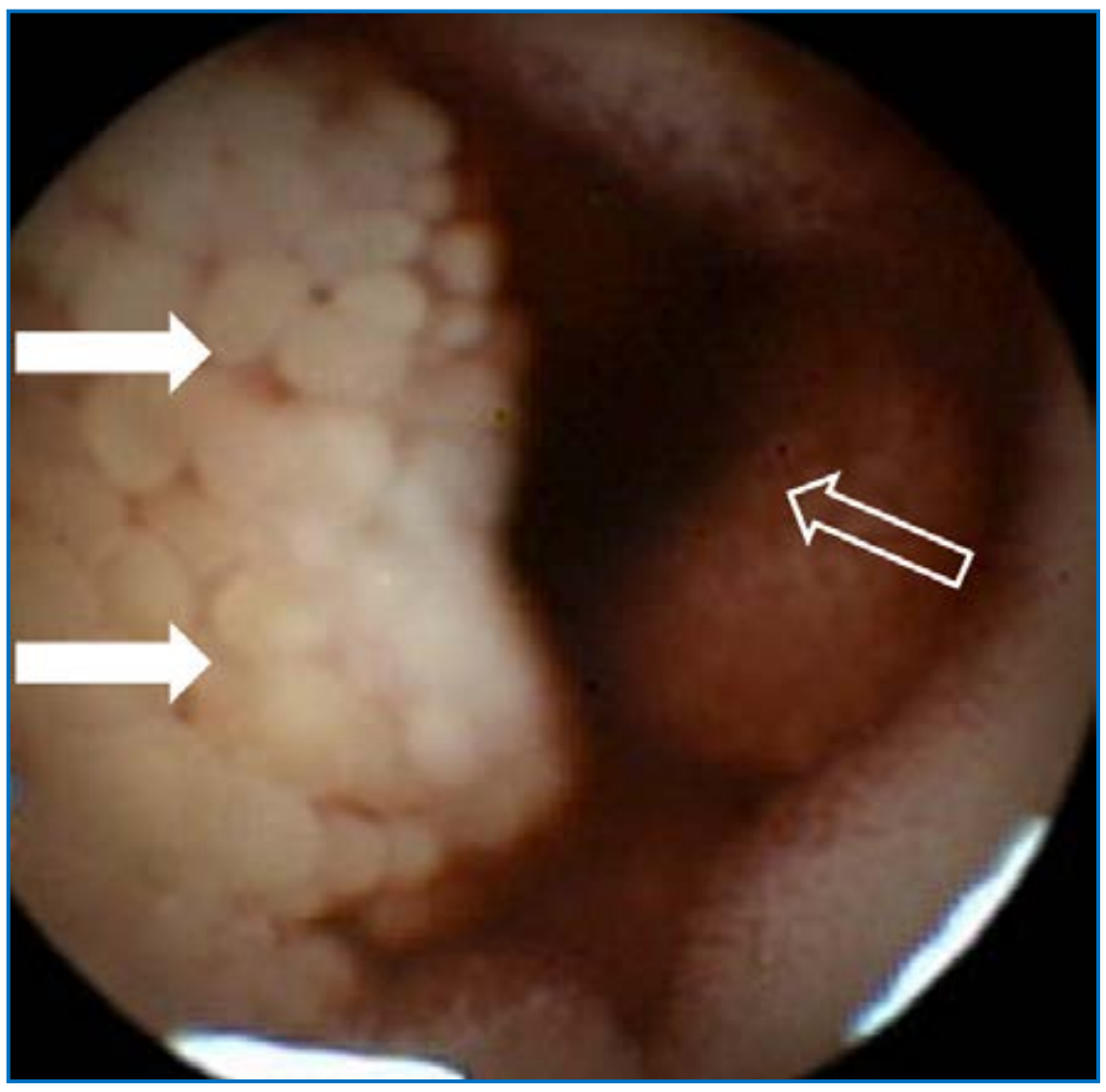

Figure 23
Small intestinal adenocarcinoma in celiac disease Marsh 3a (Marsh-Rostami-Oberhuber classification). White-light endoscopy of the distal duodenum. Circular polypoid tumour causes tight stenosis of the lumen.

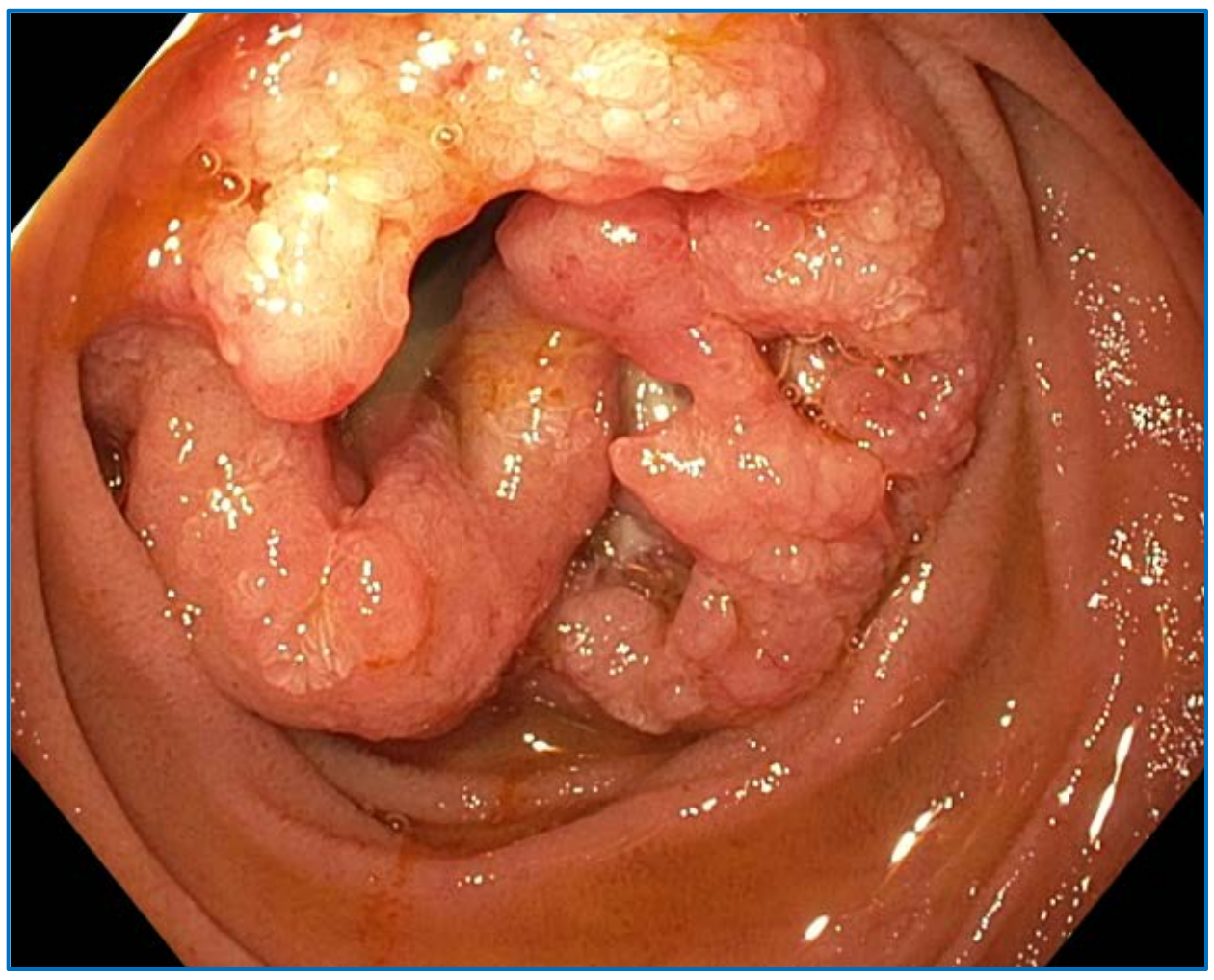

Figure 24
Postoperative bleeding. Resection of a pancreatic tumour (sec. Beger) was complicated by acute bleeding from an intestinal loop of the anastomosis. Extravasation from the lower pancreato-duodenal branch of the upper mesenteric artery is marked with open arrows. The bleeding was controlled with intra-arterial injection of histoacryl (with lipiodol).

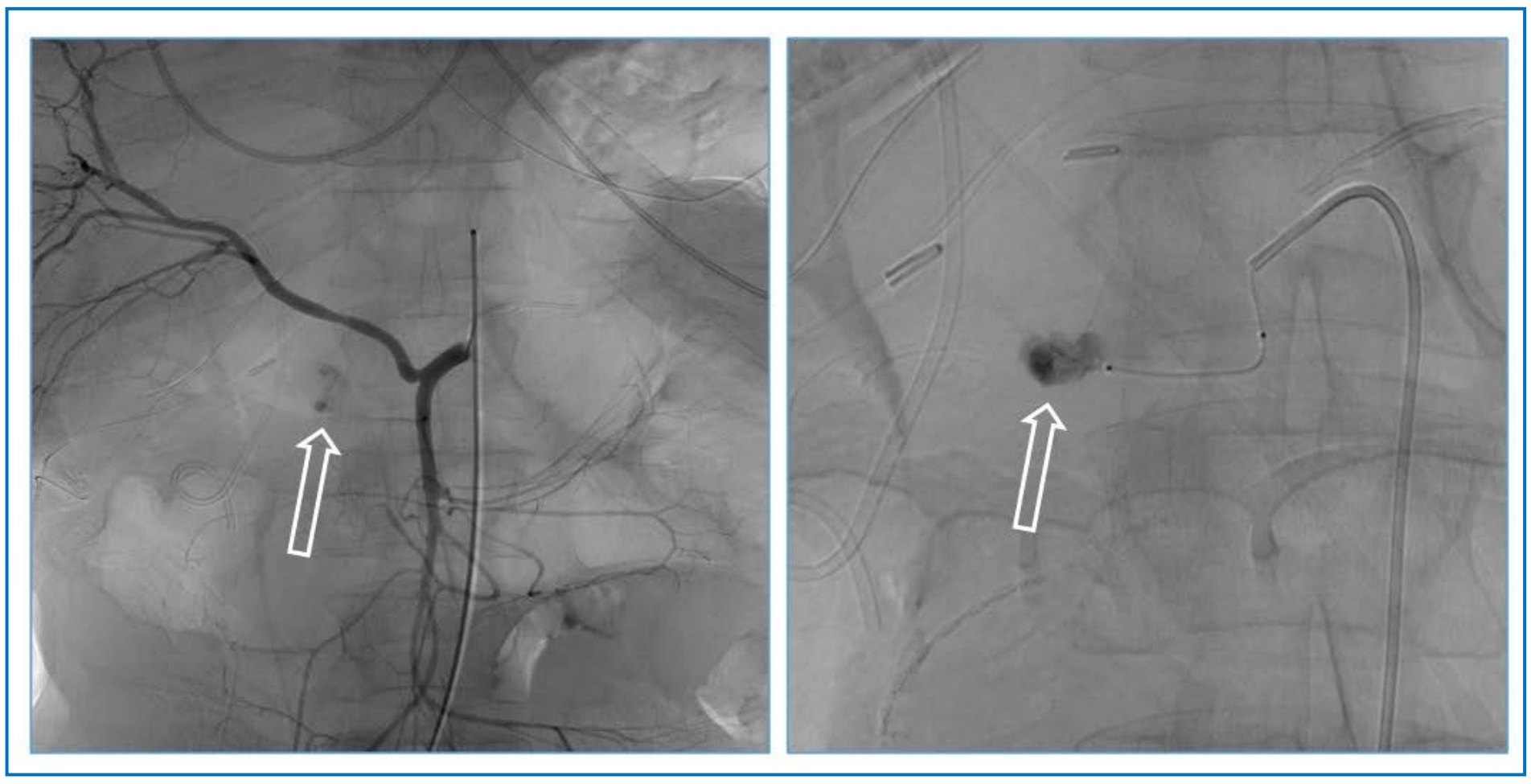

Figure 25
Functional hyposplenism. Flow cytometry of peripheral blood. Healthy blood donor on the left, dysfunction of memory B lymphocytes (CD19+ CD27+) in celiac disease on the right.

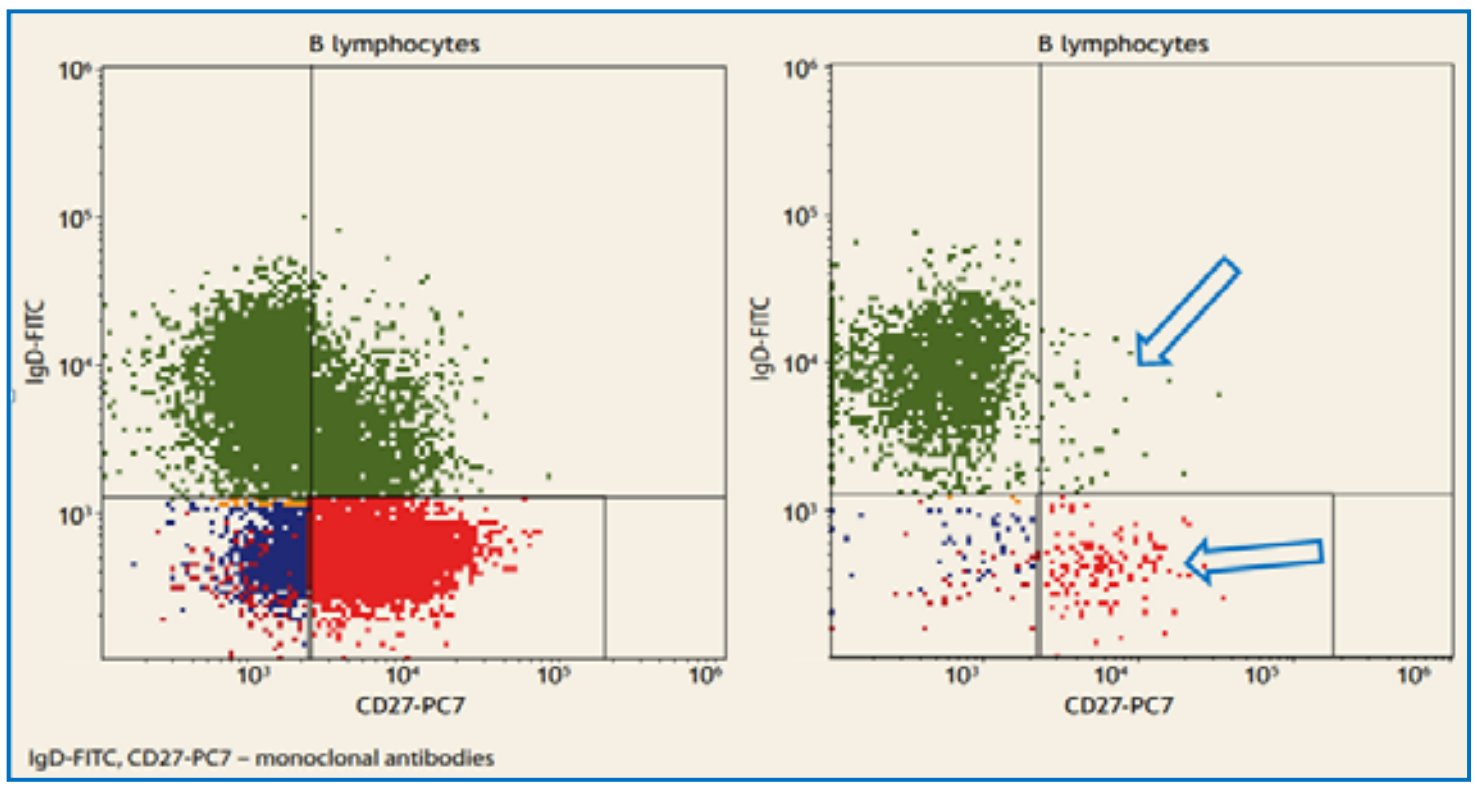

Supplement: Supplementary file 1 [file SupplementaryFile1.pdf]
